# Electroactive fiber optics for real-time, multiscale probing of ion transport and interfacial chemistry

**DOI:** 10.1038/s41377-026-02521-0

**Published:** 2026-11-09

**Authors:** Tatjana Šafarik, Frédéric Kanoufi, Médéric Loyez, Hadrien Fasseaux, David Aymé-Perrot, Nilanka M. Keppetipola, Murugasenapathi N K, Steven T. Boles, Christophe Caucheteur, Jean-Marie Tarascon

**Affiliations:** 1 Chimie du Solide et de l’Energie, UMR 8260 CNRS, Collège de France, Paris, 75005 France; 2 Université Paris Cité, CNRS, ITODYS, F-75013 Paris, France; 3 Laboratoire de Physique de l’École normale supérieure, ENS, Université PSL, CNRS, Sorbonne Université, Université Paris Cité, F-75005 Paris, France; 4 Electromagnetism and Telecommunication Department, Advanced Photonic Sensors Unit, University of Mons (UMONS), 7000 Mons, Belgium; 5 TotalEnergies SE, OneTech, 2 Place Jean Millier, La Défense 6, Paris, 92078 France; 6 Department of Energy and Process Engineering, Faculty of Engineering, Norwegian University of Science and Technology (NTNU), 7491 Trondheim, Norway

**Keywords:** Optical sensors, Imaging and sensing

## Abstract

Understanding charge dynamics at electrochemical interfaces is essential for establishing the basic mechanisms that underpin the functionality of energy storage devices and ultimately improve their performance, safety and longevity. However, direct, time-resolved access to such information remains challenging due to the complex and convoluted conditions necessary for charge transfer between materials and phases. Here, we report an electroactive fiber-optic device that integrates plasmonic sensing with electrochemical functionality, enabling real-time, multi-scale tracking of interfacial physicochemical changes. Gold-coated tilted fiber Bragg gratings were employed as dual-purpose sensors, simultaneously acting as working electrodes and optical probes of the resulting dynamics. By quantifying spectral response we demonstrate the ability to discriminate charge transfer phenomena across multiple regimes: double-layer formation, ionic and molecular adsorption, faradaic reactions involving both soluble redox species and immobilized thin solid films. By integrating a novel ‘optical-mass’ sensitivity concept, this approach unlocks access to comprehensive operando, real-time monitoring in both aqueous and organic electrolytes. As a first step toward practical deployment, we further demonstrate the feasibility of embedding a surface plasmon resonance tilted fiber Bragg grating sensor within a sealed 18650 Na-ion cell, enabling simultaneous electrochemical and optical interrogation of parasitic redox processes during formation. These results provide a new foundation for non-invasive, mechanistic studies of critical importance to energy storage, electrocatalysis and advanced materials technologies.

**Figure:**
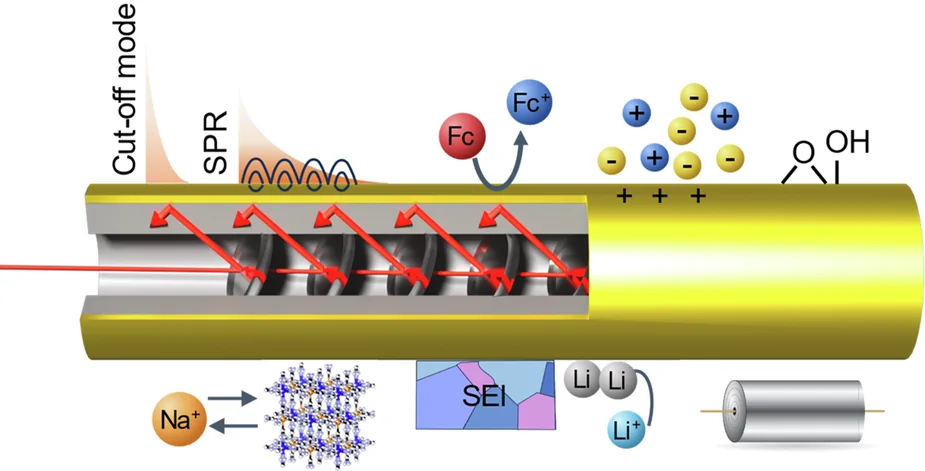

## Introduction

The accelerating need to mitigate climate change and meet the growing global energy demand is driving the continuous evolution of energy storage technologies toward safer, affordable, more reliable and sustainable solutions. Among them, rechargeable batteries are at the forefront of this transition, enabling the widespread adoption of renewable energy sources and reducing dependence on fossil fuels. However, their performance and lifetime are governed by the ever-complex interplay of driving forces and fluxes inside each cell, evidenced by concentration gradients, ionic and electronic transport, phase transformations and electrochemical reactions. Capturing these phenomena, particularly at interfaces within sealed/welded batteries, remains a major challenge for optimization, as well as safety and failure prevention.

While advanced characterization techniques such as nuclear magnetic resonance (NMR), cryo-electron microscopy, infrared (IR) spectroscopy and electron paramagnetic resonance (EPR) provide valuable insights, they often require complex instrumentation and sample preparation, limiting real-time, operando monitoring of operational systems^1–4^. Optical fibers, with diameters below 200 µm, are considered as a compact and appealing non-invasive solution to battery sensing. Their incorporation into battery packaging (i.e., around cells) enables in situ and operando monitoring, with applicability both as an in-house development tool and for widespread module-level integration^5^. In particular, fiber Bragg grating (FBG) sensors track local temperature, pressure and strain, all of which have critical links to key indicators of heat generation, volumetric deformation and gas build-up that are essential for overall battery health assessment^6^.

Despite these advances, direct chemical or electrochemical information pertaining to ion movements remains inaccessible with FBGs alone. To bridge this gap, researchers have turned to evanescent wave sensing, where light guided in and along the surface of an optical fiber interacts with the surrounding medium, enabling chemical environment sampling with negligible perturbance to the environment itself. Among such approaches, tilted fiber Bragg grating (TFBG) sensors are particularly promising: by coupling light into cladding modes, strong evanescent fields are generated at the fiber surface, which interact with near-surface physicochemical species. This allows TFBGs to sense local changes in density, temperature, refractive index (RI), composition and turbidity; facilitating assessment of electrolyte/electrode dynamics, interfacial processes and even degradation mechanisms^5,7,8^. However, their limited intrinsic selectivity for interfacial changes over near-surface (bulk) variations within the probed µm layer restricts the quantitative chemical specificity necessary for operando mechanistic studies.

This selectivity limitation is overcome by coating TFBGs with thin gold layers to enable surface plasmon resonance (SPR). This configuration enhances sensitivity ten-fold while transferring SPR’s intrinsic interfacial sensitivity to the fiber substrate: the plasmonic field confined at the metal/electrolyte boundary (sub-µm penetration) makes the optical response predominantly sensitive to interfacial RI changes, adsorption and charge accumulation rather than bulk variations^9^. Originally developed for biosensing applications^10^, SPR-TFBGs offer a compact, yet flexible, platform for monitoring electrochemical phenomena, successfully tracking supercapacitor state of charge^11^ and ion fluxes in Zn-ion batteries^12^. However, in these earlier implementations, the fiber was generally positioned passively ‘near’ the electrode to be probed, with limited control of its exact position. This constraint is particularly critical when aiming to resolve localized electrode phenomena such as ion insertion, solid-state conversion reactions, lithium plating, steep concentration gradients, or interfacial adsorption processes such as solid electrolyte interface (SEI) formation^13–15^.

Herein, we demonstrate an integrated configuration where the gold-coated SPR-TFBG fiber simultaneously serves as both the working electrode and optical probe, directly addressing the spatial localization challenge noted above. This design, inspired by electrochemical SPR (EC-SPR) systems with planar electrodes^16–18^, offers a miniaturized alternative for remote or embedded applications. Unlike prior electrochemical implementations that exploited the electrode function to electrophoretically concentrate biomolecular targets^19^ or to accumulate metal ions for detection^20^, the present work turns this dual-function architecture toward mechanistic interrogation of the electrochemical interface itself: the SPR-TFBG simultaneously resolves interfacial processes that govern energy-storage operation (double-layer charging, specific adsorption, interphase formation, redox, underpotential deposition, alloying and ion intercalation) via the SPR mode and the bulk contributions via cut-off modes, quantifying them against charge through the $\Delta {\lambda }_{{{{\rm{SPR}}}}}-\,\Delta {Q}$ relationship (“optical voltammetry”). To our knowledge, this is the first use of the electrode-integrated SPR-TFBG for operando, charge-resolved study of battery-relevant interfacial chemistry in organic electrolytes and within a sealed 18650 cell.

As a proof of concept, we apply this monitoring platform to three exemplary cases: 1) double-layer and gold oxide formation, and ferrocenemethanol reduction in aqueous media, 2) native SEI evolution on Au in non-aqueous media, and 3) Na(K)-driven redox in Prussian blue electrodes. Concerning data treatment, we implement a demodulation method based on Fourier transform (FT) analysis of the SPR signature^9,21^, exploiting complementary cladding modes for refractive-index-sensitive readouts of near-surface bulk contributions. Taken together, these elements demonstrate the multimodal nature of SPR-TFBG sensors, enabling surface-specific interfacial (SPR-based) and near-surface bulk (cut-off-based) monitoring of electrochemical interfaces across multiple length scales of interest.

As demonstrated in this work, the SPR mode achieves picometer-level wavelength resolution, corresponding to an apparent thickness sensitivity of only few picometers, equivalent to detecting (optical) mass changes on the order of ~10^2 ^pg cm^-2^. This extreme surface sensitivity can thus provide a unique window for simultaneously viewing both interfacial and bulk processes with effectively monolayer resolution. Such analytical capabilities have broad implications, not only for similar systems including non-Faradaic capacitors and electrocatalytic devices, but also for advancing new, holistic perspectives of interfacial charge transfer and molecular-scale dynamics in a range of humanity critical systems, from photovoltaics to water-splitting technologies.

## Results

### Key features of SPR-TFBG sensing

**Fig. 1:**
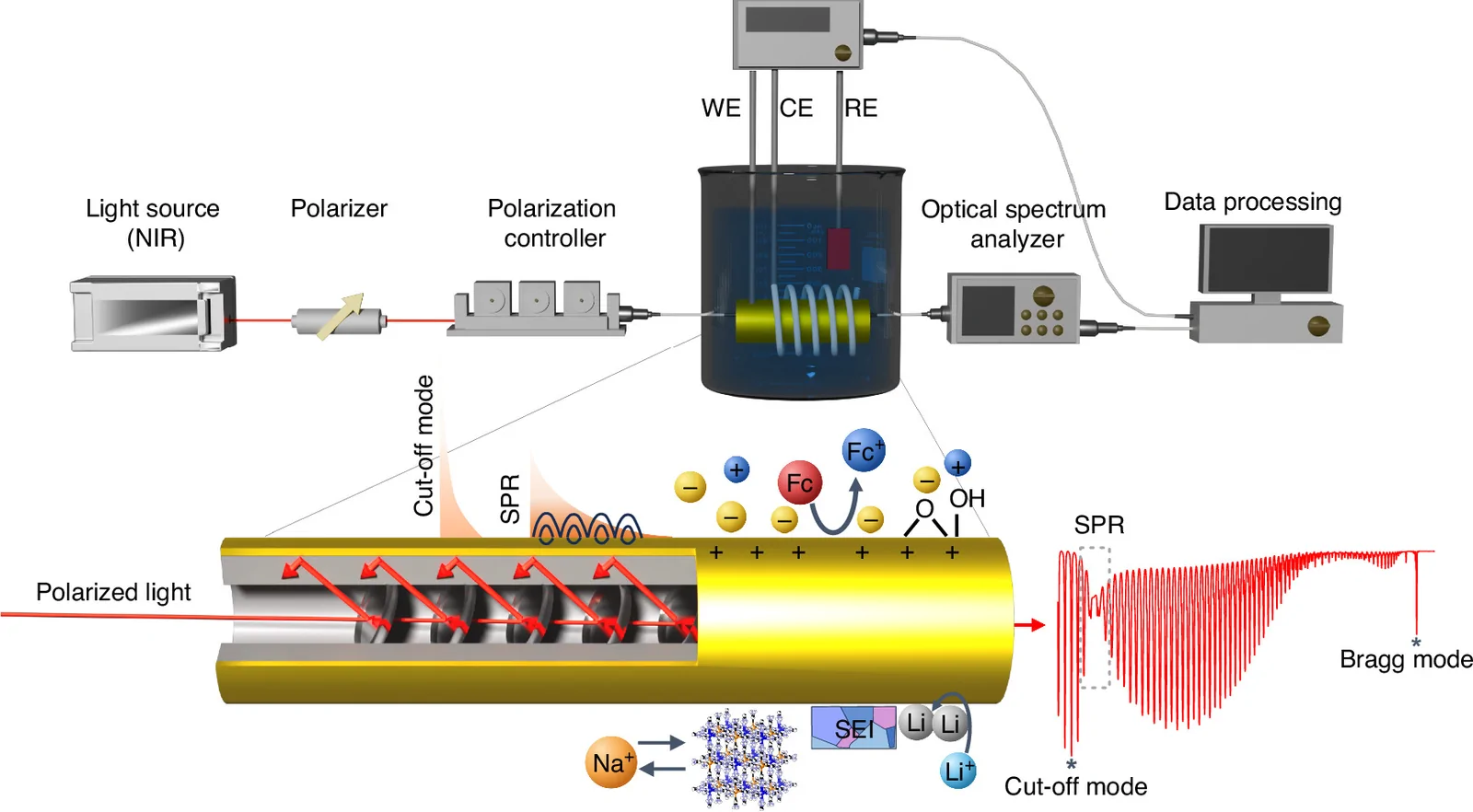
**Experimental setup and sensing principle**. A gold-coated tilted fiber Bragg grating (TFBG) is connected in transmission between a broadband near infrared (NIR) source and optical spectrum analyzer, with an in-line polarizer (only P-polarized light excites surface plasmons) following the NIR source. The gold coated TFBG fiber also serves as the working electrode (WE) in a three-electrode electrochemical configuration, together with counter (CE) and reference (RE) electrodes connected to a potentiostat. The enlarged view (lower left) shows the TFBG structure imprinted into a single-mode 125 μm fiber and coated with a 50 nm gold film, together with a schematic representation of the sensing depth: the cut-off mode probes the near-surface bulk, whereas SPR probes the interface (the first few nanometers). Icons indicate electrochemical processes: double-layer charging, ferrocenemethanol (Fc) redox, gold oxide formation, SEI formation, lithium plating, and Prussian blue electrochemistry. A representative transmission spectrum, measured in water (lower right), shows SPR resonance (dip), cut-off and the Bragg mode positions

To probe multi-scale transport and chemical phenomena, consideration must be given to the placement, sequence and arrangement of numerous essential system components. Figure 1 presents the experimental configuration, where the sensor primarily consists of a TFBG inscribed in a single-mode optical fiber (SMF) and interrogated in transmission. The TFBG operating principle is briefly outlined, as it underpins our strategy for monitoring electrochemical processes at the nanoscale (see Supplementary Information for more detail, or earlier works^5,7^). Unlike standard fiber Bragg gratings, TFBGs have grating planes tilted relative to the fiber axis, coupling core-guided light into cladding modes whose evanescent fields extend beyond the fiber surface and interact with the surrounding medium. Each cladding mode corresponds to a specific resonance wavelength, producing the oscillatory transmission shown in Fig. 1. The highest refractometric sensitivity is achieved near the cut-off wavelength (marked *), where the mode’s effective RI matches the surrounding medium. TFBG responses are tracked through wavelength shifts (nm) and/or amplitude changes (dB)^5,7^. Because wavelength shift tracking is more robust against unwanted optical power (i.e., intensity) fluctuations, it is selected here as the primary indicator of the optical responses throughout this study.

TFBGs were conformally coated with a thin gold layer (<50 nm) by sputter deposition, providing electrical conductivity for electrochemical operation while enabling SPR excitation. Plasmon excitation appears as a sharp attenuation (plasmonic dip due to energy transfer from specific cladding modes to surface plasmons) in the transmission spectrum over a narrow wavelength region (labeled SPR in Fig. 1). The gold-coated SPR-TFBG was used directly as the working electrode in a conventional three-electrode cell, with a platinum counter and an Ag/AgCl reference electrodes. The optical response was recorded simultaneously with electrochemical actuation under galvanostatic control, cyclic voltammetry, or at open-circuit potential. Changes in cut-off and SPR resonance wavelengths tracked interfacial processes at different length scales, while the Bragg mode provided an internal reference sensitive only to strain and temperature.

The multimodal SPR-TFBG response offers rich spectral information but presents nontrivial data processing challenges. On one hand, the ~5 s spectral acquisition rates employed are sufficient for capturing much of the essential solid-liquid interfacial dynamics that govern common electrochemical devices. Additionally, as compared to prism-based SPR systems, the fiber configuration affords more complex integration into confined or opaque environments, still within the bounds of 1D planar models and approximations. However, to reliably track SPR wavelength shifts, we implemented Fourier transform (FT) demodulation^9,21^, providing improvement in sensitivity and signal-to-noise ratio compared to traditional single-resonance tracking (Fig. S1). In contrast, single-resonance tracking was used for the cut-off mode, which lacks a distinct spectral signature and therefore does not benefit from FT analysis.

Despite these differences, both modes respond similarly to bulk refractive index, RI, variations in aqueous K_2_SO_4_. This is illustrated in Fig. S2, where increasing the electrolyte concentration from 0.1 to 0.5 M induces a comparable increase in resonance wavelength, later denoted as a red shift, for both SPR and cut-off modes. The linear dependence of the red shift on the bulk RI (*R*^2^ > 0.999) yields a sensitivity of 577 ± 1 nm RIU^−1^ for both modes.

These behaviors are consistent with a classical description of evanescent field behavior for optical sensing of a surrounding medium. For both SPR and cut-off modes the wavelength shifts, $\Delta \lambda$, associated with local changes in RI, Δn, in the surrounding medium can be expected to behave according to the following relation:

1 $$\Delta \lambda =m{\int }_{0}^{\infty }\Delta n(z)\exp \left(-\frac{z}{{l}_{{{{\rm{d}}}}}}\right)\frac{{{{\rm{d}}}}z}{{l}_{{{{\rm{d}}}}}}$$

with m the bulk sensitivity (m = 577 nm RIU^−1^ for K_2_SO_4_), z the distance from the electrode surface in the solution, Δn(z) the refractive index change along z (possibly varying with electrochemically induced concentration gradients) and ${l}_{{{{\rm{d}}}}}$ the characteristic decay or penetration length of the evanescent field, in the sub-µm to µm range^12,22^ for SPR and the cut-off mode respectively. Generally, the local refractive index change $\Delta n\left(z,t\right)$ is computed from the concentration profiles using:

2 $$\Delta n\left(z,t\right)={\sum}_{i}{\left(\frac{{{{\rm{d}}}}n}{{{{\rm{d}}}}c}\right)}_{i}\Delta {c}_{i}(z,t)$$

with ${\left(\frac{{{{\rm{d}}}}n}{{{{\rm{d}}}}c}\right)}_{i}$ the RI change with concentration and $\Delta {c}_{i}(z,t)$ the local change in concentration of species i. When the concentration is uniform over distances, so is the RI, $\Delta n\left(z\right)=\Delta n$, and the expression simplifies to $\Delta \lambda =m\Delta n$ explaining why both SPR and cut-off modes show nearly identical linear responses to bulk RI changes (Fig. S2).

### Electrooptical measurement of the Au/electrolyte interface

**Fig. 2:**
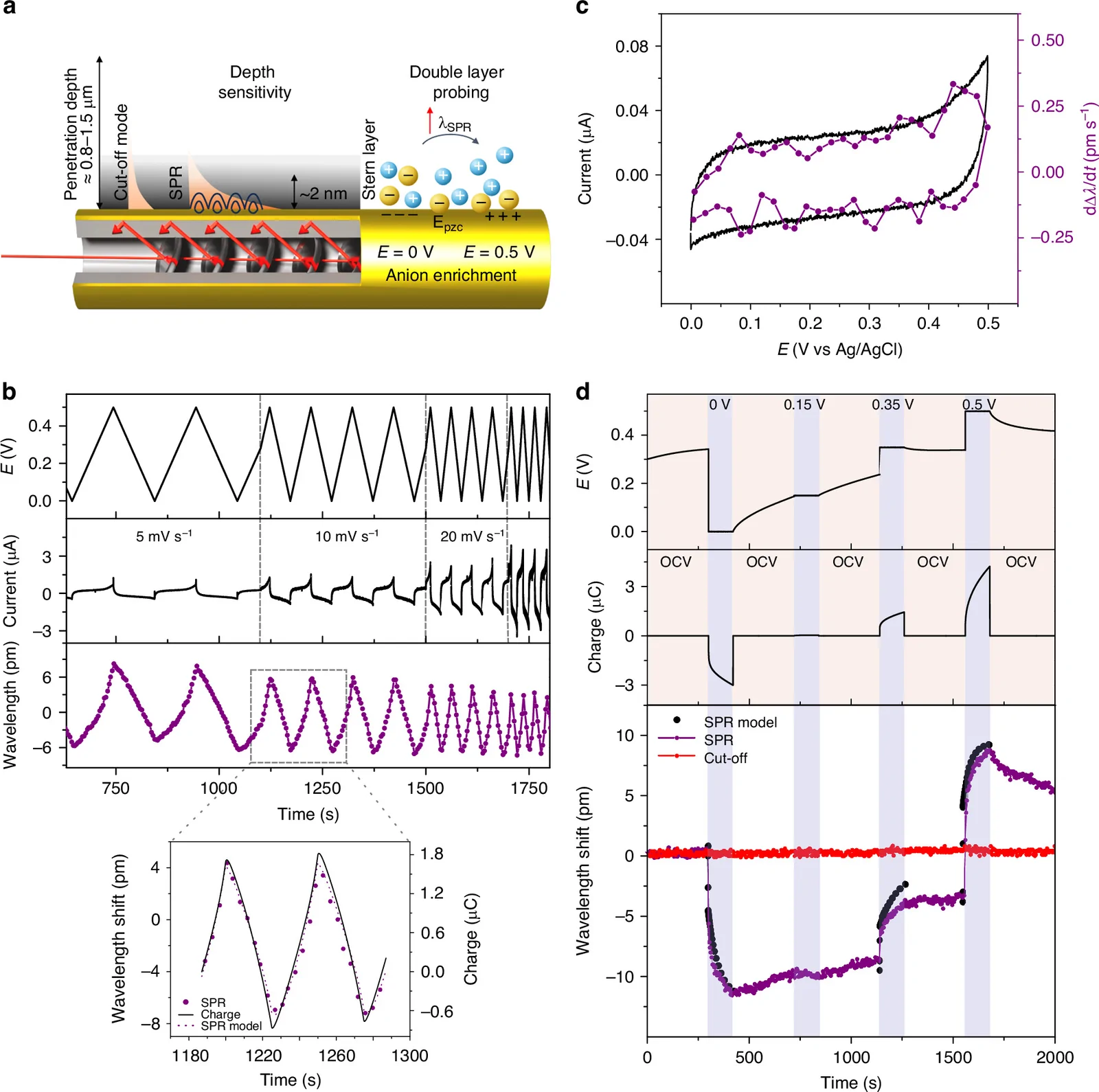
**Optical probing of electrochemical double-layer charging at a gold-coated SPR-TFBG electrode**. **a** Schematic comparison of the depth and surface sensitivity of the SPR and cut-off modes at a gold-coated SPR-TFBG electrode, shown relative to the ~2 nm compact electrochemical double layer (EDL). **b** Electrode potential (E), current and SPR wavelength shift ($\Delta \lambda$, purple symbols) recorded during cyclic voltammetry of the gold electrode at scan rates 5, 10, 20, 35 mV s^−1^ (consecutive repeated cycles) in 0.1 M K_2_SO_4_. The zoomed region (bottom) compares, over two cycles at 10 mV s^-1^, the measured SPR wavelength shift (symbols) with the electrochemical charge (black line) and the modeled SPR response (purple dashed line) obtained from Eq. (4), using the computed ionic distribution in solution and DL and SO_4_^2−^ adsorption/desorption at the gold electrode. **c** Cyclic voltammogram (capacitive current) overlaid with the first-order derivative of the SPR shift ${{{\rm{d}}}}\Delta \lambda /{{{\rm{d}}}}t$, scan rate 5 mV s^−1^. **d** Sequential potential steps (0, 0.15, 0.35 and 0.5 V vs Ag/AgCl; 2 min each) followed by 5 min open circuit relaxation in 0.1 M K_2_SO_4_: applied potential (top), charge (middle) and wavelength shifts measured in SPR (bottom, purple symbols, black line: model) and in cut-off mode (red symbols)

We next benchmark the optical responses with fundamental interfacial electrochemical processes. Double-layer charging (DLC) at the Au/electrolyte interface was examined in 0.1 M K_2_SO_4_ while recording transmission spectra to analyze SPR and cut-off modes contributions separately (schematically illustrated in Fig. 2a). The capacitive nature of the interface is directly evidenced from cyclic voltammetry (CV) experiments between 0 and 0.5 V (vs Ag/AgCl) at scan rates ranging from 5 to 50 mV s^−1^ presented as the variation of the applied potential and measured current in Fig. 2b (see Figs. S3–S5 for other scan rates and supporting analyses). The integrated charge, presented in the zoomed-in portion of the 10 mV s^−1^ CV (Fig. 2b), varies as anticipated with the applied potential, tracking charge flows during anodic and cathodic scan periods. Simultaneously during these scans, the SPR resonance (lower panel and zoomed portion of Fig. 2b) undergoes red and blue shifts during anodic and cathodic polarization, respectively, thus following the reversible charging and discharging of the Au/electrolyte interface (zoomed region of Fig. 2b). Closer scrutiny reveals a strong linear correlation between the electrochemical charge accumulated and the SPR wavelength shift (Figs. S4 and S5).

Comsol simulations based on the Poisson-Nernst-Planck formalism (discussed in the SI, including further context from the following section regarding FcMeOH), were used to compute the electrolyte ion redistribution in solution and near the electrode during electrochemical polarization. The resulting change in bulk RI attributed to the current flow is negligible, corresponding to an expected wavelength shift of <0.1 pm according to Eq. (1). This agrees with the absence of movement in the cut-off signal and confirms that the SPR mode probes interfacial phenomena in addition to bulk RI changes. Perturbations in the SPR mode signal may then also stem from fine scale phenomena, such as the rearrangement of ions in the electrical double layer (EDL), a consequence of compensation for the electrochemical charge, ΔQ. The SPR response can therefore contains an additional term written as:

3 $$\Delta \lambda ={\kappa }_{{{{\rm{\sigma }}}}}\Delta Q$$

where ${\kappa }_{{{{\rm{\sigma }}}}}$ is the interfacial optical sensitivity to electrochemical charge and ΔQ the change in DL charge.

Approximating the DL as a constant capacitance ${C}_{{{{\rm{dl}}}}}$ a linear sweep of potential $E\left(t\right)={vt}$ at scan rate v gives $\Delta Q={C}_{{{{\rm{dl}}}}}E={C}_{{{{\rm{dl}}}}}{vt}$, where t is the elapsed time during the charge/discharge process. Equation (3) accounts for the observed linear relationship between the SPR wavelength shift and electrochemical charge, from which the SPR charge sensitivity is estimated ${\kappa }_{{\rm\sigma }}=4.75\,{{\mathrm{pm}}}\,{{\rm\mu }{{{\rm{C}}}}}^{-1}$ (Fig. S5). Because $\Delta \lambda$ is proportional to$\,\Delta Q$, differentiation gives$\,\frac{{{{\rm{d}}}}\Delta \lambda }{{{{\rm{d}}}}t}\propto \frac{{{{\rm{d}}}}Q}{{{{\rm{d}}}}t}$ making the time derivative of the SPR signal directly proportional to the electrochemical current, conveniently allowing for the straightforward comparison of $\frac{{{{\rm{d}}}}\varDelta \lambda }{{{{\rm{d}}}}t}$ traces with voltammograms (as confirmed in Fig. 2c). Notably, for capacitive charging/discharging of the Au/electrolyte interface, the cut-off response remains negligible, emphasizing that SPR is highly selective to surface-localized processes, while the cut-off mode is largely insensitive to them.

Upon decreasing the scan rate (Fig. S4) the total charge stored in the double layer slightly increases, by ~0.8 µC, again correlating with an increase in SPR wavelength shift (Fig. 2b). This excess charge, beyond pure double-layer capacitive contribution, reflects additional interfacial reorganization such as specific adsorption of sulfate species. Such adsorption/desorption processes at the Au interface can be further included through an additional term:

4 $$\Delta \lambda ={\kappa }_{{{{\rm{\sigma }}}}}\Delta Q+{\kappa }_{\Gamma }\Delta {\Gamma }_{{{{\rm{ads}}}}}$$

with ${\kappa }_{\Gamma }$ the optical sensitivity to surface-adsorbed species and $\Delta {\Gamma }_{{{{\rm{ads}}}}}$ the change in adsorbate surface concentration. Here, the optical sensitivity for adsorbed species reflects the RI or polarizability changes associated with the processes of attaching to or leaving the Au surface, and the SPR wavelength shift is thus expected to increase (red shift) upon species adsorption and decrease (blue shift) upon species desorption.

Such slow, interfacial (re)structuring dynamics of the Au surface are more clearly observed under potentiostatic steps from 0 to 0.5 V followed by 5-min open circuit relaxation (OCV), where the long timescale optical evolution (Fig. 2d) again shows a static cut-off mode while the SPR $\Delta \lambda$ continues to track and measure the dynamics of surface localized electrochemical charges. Stepping the potential from OCV to 0 V produces a quasi-instantaneous ca. -7 pm blue shift, followed by a slower (minute-scale) blue shift of ca. -6 pm, along with slow negative charging of the electrode (Figs. S6 and S7), consistent with depletion or desorption of interfacial sulfate. In contrast, the step from OCV to 0.5 V yields a pronounced red shift (positive $\Delta \lambda \approx \,8\,{{{\rm{pm}}}}$) which continues with more mild red shifting thereafter, by ca. 6 pm, as slow positive charging of the electrode proceeds (Figs. S6 and S7), over several tens of seconds. This slow evolution reflects the progressive accumulation and rearrangement of specifically adsorbed sulfate and associated counter cation and hydration layers as the potential moves further above the point of zero charge (pzc).

Upon returning to OCV, $\Delta \lambda$ relaxes back gradually (noted after 0 and 0.5 V) following the variation of the measured open circuit potential (Fig. 2d top panel). The SPR wavelength shift, as well as the electrode potential, reflects the slow and partial desorption (or re-adsorption after 0 V step) of the interfacial structure. The overall symmetric response upon stepping to 0 or to 0.5 V highlights the sensitivity of the SPR mode to potential-dependent adsorption equilibria and interfacial restructuring.

Although quantitatively predicting these processes requires knowledge of the detailed adsorption isotherm and dynamics, COMSOL simulations that combine DL charging and a simple potential-dependent adsorption process (see details in SI and Fig. S8) successfully reproduced the overall trends of the CV (zoomed region of Fig. 2b for 10 mV s^−1^, other scan rates in Fig. S8a) and potentiostatic experiments (dashed lines in the lower panel of Fig. 2d). A limited sensitivity analysis of the main model parameters is provided in Fig. S8c,d showing the impact of adsorption dynamics on the simulated behavior. Reproducing the experimentally observed variations from the model enables estimating the pzc, where net surface charges are minimized and SPR shift is zero. In this particular system, it is evaluated to be 0.24 V in agreement with reported values^23^. The potential-dependent kinetics of the slow SPR shift is evaluated (on the order of 3 × 10^−3 ^s^−1^ at pzc) and shown to be specific to sulfate accumulation and release at the Au surface.

These results demonstrate the ability of the SPR-TFBG sensor to detect interfacial reorganizations at the sub-nanometer scale, including slow adsorption and desorption phenomena at the Au/electrolyte interface. However, the processes discussed involve non-Faradaic processes limited to the electrical double layer and its immediate vicinity. While likely useful for some electrochemical applications, such as with electrode stability in capacitive deionization^24^, we seek to push the method further and explore interfacial reactivity beyond the electrochemical DL. Since electron and ion transfer are often coupled in electrochemical systems yet governed by different factors, we aim to explore Faradaic processes, probe molecular-scale redox reactions and transformations where mass transport from the bulk solution adds an essential layer of complexity.

### Probing redox processes and diffusion layer

**Fig. 3:**
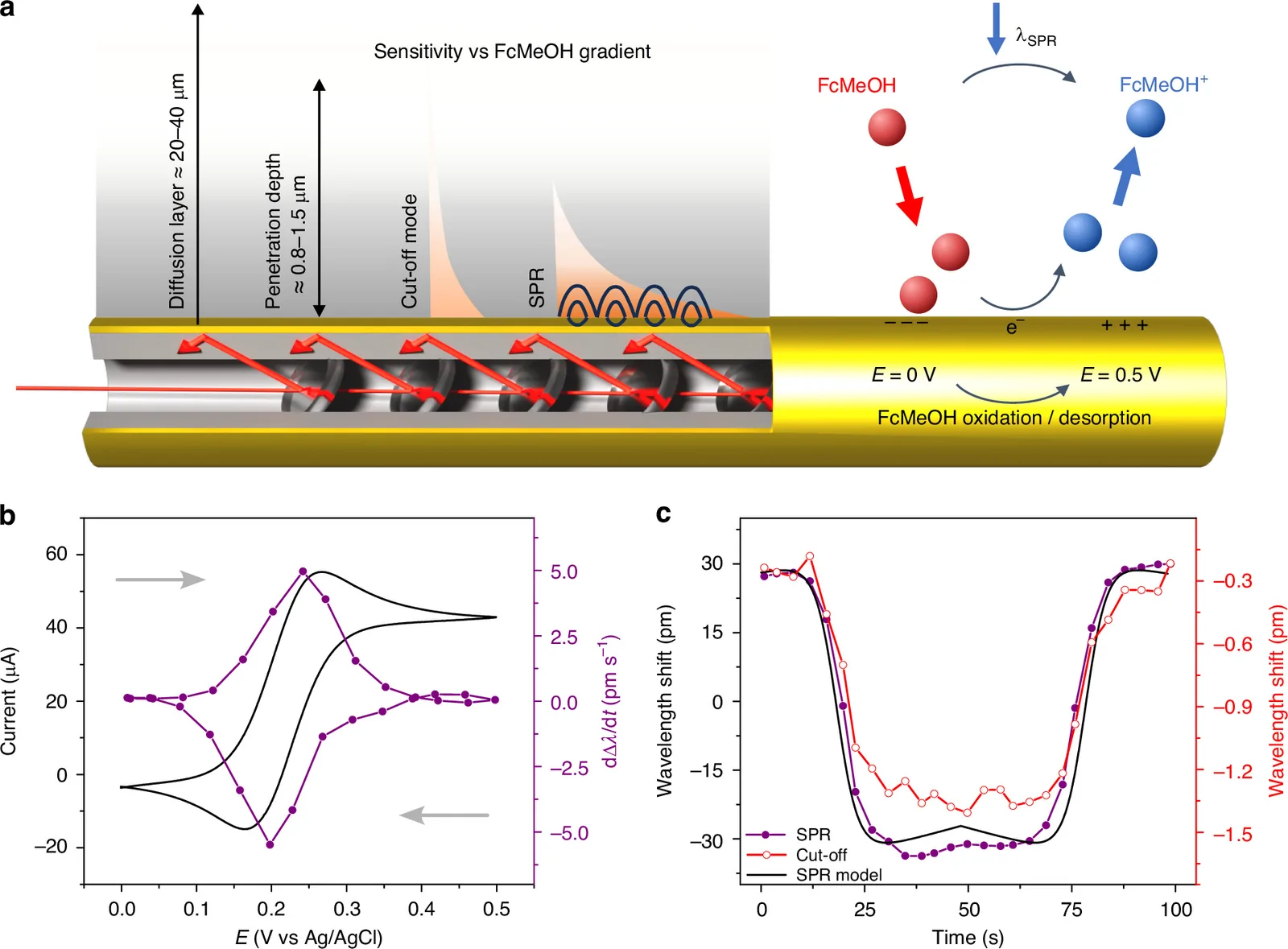
**Electrooptical measurement of Au/electrolyte interface during the FcMeOH redox cycle**. **a** Schematic of Au/electrolyte interfacial restructuring during ferrocenemethanol (FcMeOH) oxidation-reduction cycle on the fiber: desorption/adsorption of ferrocene species, near-surface distribution and formation of diffusion layer whose thickness contrasts with the optical penetration depths and the surface selectivity of SPR. **b** Cyclic voltammogram of 5 mM FcMeOH in 0.5 M K_2_SO_4_ at 10 mV s^−1^ (black trace) overlaid with the time derivative of the SPR wavelength shift (${{{\rm{d}}}}\Delta \lambda /{{{\rm{d}}}}t$, purple symbols). **c** Experimental wavelength shifts measured in SPR (purple symbols) and in the cut-off mode (red symbols) during the CV in (**b**), showing the sigmoidal temporal response. The black line is the modeled SPR shift obtained from COMSOL simulations (SI) and Eq. (6)

To probe interfacial dynamics involving both electron transfer and mass transport, we employed ferrocenemethanol (FcMeOH), a well-known outer-sphere redox couple which undergoes reversible electron exchange at the Au electrode surface (Fig. 3a). CV scans recorded in 5 mM FcMeOH in 0.5 M K_2_SO_4_ and monitored by SPR-TFBG sensors (Fig. 3b) display peak-shaped voltammograms with currents varying as the square root of the scan rate (Fig. S9), as expected from the Randles-Ševčik equation for reversible redox systems. Unlike DLC, redox reactions constantly consume species at the reaction site/surface, generating concentration gradients that extend far into the electrolyte. For FcMeOH, which has a diffusion coefficient of $D\,=\,7.5\,\times \,{10}^{-6}\,{{{{\rm{cm}}}}}^{2} \,{{{{\rm{s}}}}}^{-1}$ the diffusion layer thickness δ during CV at scan rate ν evolves as $\delta =\sqrt{\frac{{DRT}}{{Fv}}}$ for $\nu =10-50 \,{{{\rm{mV}}}}{{{{\rm{s}}}}}^{-1},\,\delta$ varies between 20 and 45 µm, about 20 times greater than the optical penetration depth^25^. Thus, for the first micrometer from the electrode surface, the concentrations of FcMeOH and FcMeOH^+^ can therefore be approximated as quasi-uniform and equal to their interfacial values. In this limit, the wavelength shifts of both modes reduce to $\Delta \lambda =m\Delta {n}_{{{{\rm{eff}}}}}$ with $\Delta {n}_{{{{\rm{eff}}}}}$ the effective RI change associated with the FcMeOH/FcMeOH^+^ conversion. A full expression, in agreement with an earlier report^18^, including electroneutrality contribution from supporting electrolyte species is computed from Eq. (2). The dominant term reduces to a balance between the RI contributions of FcMeOH and FcMeOH^+^:

5 $$\Delta {n}_{{{{\rm{eff}}}}}=\,\left[{\left(\frac{{{{\rm{d}}}}n}{{{{\rm{d}}}}c}\right)}_{{{{{\rm{Fc}}}}}^{+}}-{\left(\frac{{{{\rm{d}}}}n}{{{{\rm{d}}}}c}\right)}_{{{{\rm{Fc}}}}}\right]{\Delta \left[{{{{\rm{FcMeOH}}}}}^{+}\right]}_{{{{\rm{el}}}}}$$

where ${\left({{{\rm{d}}}}n/{{{\rm{d}}}}c\right)}_{{{{\rm{X}}}}}$ is the change in RI per concentration unit of species X and [X]_el_ is its concentration at the electrode surface.

During the anodic scan of the CV, both SPR and cut-off resonances undergo a blue shift (negative $\Delta \lambda$), which is reversed on the cathodic scan (Fig. 3c and Fig. S9b), indicating that both modes respond to the local depletion of neutral FcMeOH and formation of its oxidized form FcMeOH^+^ in the solution phase. According to the expression for how RI changes, the experimentally observed blue shift ($\Delta \lambda < 0$) during oxidation (${\Delta \left[{{{{\rm{Fc}}}}}^{+}\right]}_{{{{\rm{el}}}}} > 0$) therefore implies ${\left({{{\rm{d}}}}n/{{{\rm{d}}}}c\right)}_{{{{{\rm{Fc}}}}}^{+}} < {\left({{{\rm{d}}}}n/{{{\rm{d}}}}c\right)}_{{{{\rm{Fc}}}}}$, consistent with the lower polarizability of the cationic FcMeOH^+^ relative to neutral FcMeOH. This simple near-surface refractometric picture rationalizes the similar potential dependence and sign of both the SPR and cut-off responses (supported further with the simulated cut-off response in Fig. S8b).

As discussed previously for DLC, the derivative signal ${{{\rm{d}}}}\Delta \lambda /{{{\rm{d}}}}t$ should mirror the electrochemical current. As seen in Fig. 3b, the ${{{\rm{d}}}}\Delta \lambda /{{{\rm{d}}}}t$ variation during the CV indeed exhibits peaks that qualitatively track the Faradaic currents, as expected from Eq. (5) for the evolution with E of the FcMeOH species concentration at the electrode (see simulated response in Fig. S8b). To quantify this behavior, the SPR wavelength shifts were fitted with a sigmoidal function (Fig. S10), characterizing the reduced and oxidized states (and their transitions) at the electrode, during electron transfer. The fitted half-wave potential $({E}_{1/2}\approx 200\,{\mathrm {mV}})$ of the sigmoidal$\,\Delta \lambda -E$ curve coincides with the half-wave potential from the current–potential curve. The transition time between states (~3 s) is close to the 2.5 s expected for a purely Nernstian system at this scan rate, supporting fast electron-transfer kinetics^26^.

However, owing to the same refractometric sensitivity of both modes, Eq. (1) suggests similar wavelength shifts for SPR and cut-off modes. In practice, the SPR shift during cycling (~60 pm in Fig. 3c) is 60 times larger than the cut-off one (~1 pm, Fig. 3c, model in Fig. S8b), pointing to an additional signal contribution from interfacial FcMeOH adsorption/desorption in the SPR response. A limited sensitivity analysis of the main model parameters is provided in Fig. S8e-h showing the effect of adsorption and diffusion layer thickness on SPR response, though without implying a unique microscopic parameter set and not clear cut between adsorption or bulk contribution. The interpretation of SPR being dominated by surface effect is rather supported by the other surface-confined or thin-layer processes examined in this work (see below), namely LiBOB-derived interphase formation and Prussian blue deposition/conversion, for which the cut-off response is again 20–60 times smaller than the SPR response. The fact that FcMeOH exhibits the same range of SPR/cut-off contrast supports the conclusion that its optical response is also dominated by a surface-localized contribution rather than by bulk diffusion alone.

As introduced for DLC, the SPR response to FcMeOH electrochemistry should include considerations as noted in Eq. (6), stemming from changes in surface coverage of neutral and oxidized FcMeOH species, $\Delta \Gamma$ and ${\kappa }_{\Gamma }$ as these parameters reflect respective polarizabilities.

6 $$\Delta {\lambda }_{{{{\rm{SPR}}}}}=m\Delta {n}_{{{{\rm{eff}}}}}+{{\kappa }_{\Gamma }^{{{{\rm{Fc}}}}}\Delta \Gamma }_{{{{\rm{Fc}}}}}+{{\kappa }_{\Gamma }^{{{{{\rm{Fc}}}}}^{+}}\Delta \Gamma }_{{{{{\rm{Fc}}}}}^{+}}$$

Critically, the data collected indicate that the DLC and sulfate adsorption contributions can be safely neglected here, as Fig. 2 shows that their optical signatures are at least an order of magnitude smaller and of opposite sign. Given the high polarizability of FcMeOH and its reported tendency to adsorb on Au surfaces^27–29^, we attribute the enhanced SPR response mainly to potential-driven desorption of the adsorbed neutral FcMeOH upon its oxidation to FcMeOH^+^ (and its re-adsorption upon the reverse reduction scan). This is supported by the reproduction of the experimental SPR shifts by a COMSOL model depicting the adsorption/desorption of FcMeOH on the sensor (see black line in Fig. 3c).

The dynamical aspects of the potential-driven process of FcMeOH desorption are confirmed from the SPR response measured during potentiostatic experiments (Fig. S11), where the potential is stepped above ${E}_{1/2}$(0.35 and 0.5 V). Similar to the case of the DLC experiments, presented in Fig. 2d, these steps are shown to produce a rapid SPR blue-shift followed by a slower additional shift, over tens of seconds, consistent with potential-accelerated desorption dynamics. During subsequent OCV holds, the SPR signal quickly relaxes back toward the reduced-state baseline on a comparable timescale, reflecting the slow re-equilibration of FcMeOH surface coverage coupled to diffusion-controlled replenishment from the bulk.

### Optical multimodal response to gold oxide formation

The conclusions drawn above on model systems and supported by simulations also hold for more complex surface-confined solid-state transformation, such as gold oxidation in 0.5 M H_2_SO_4_, presented in SI Fig. S12. Cyclic voltammetry shows the classical sequence of gold hydroxide/oxide formation and subsequent reduction. The SPR wavelength shift exhibits a large multistep red shift during the anodic scan (on the order of several hundred picometers) and a partial blue shift upon reduction (Fig. S12) consistent with the growth and partial removal of a hydrated Au oxide layer.

In contrast, the cut-off mode shows only a small (few picometers) shift, opposite sign to SPR, at the onset of the oxide formation. However, the two signals seem to retrace right before oxide formation (~stage III) and this congruency remains during oxide reduction. The cut-off response might reflect coupled changes in near-surface electrolyte composition, including local acidification and sulfate protonation, while SPR response arises from the interfacial conversion of Au into Au oxide and associated adsorbed oxygen-containing species.

Overall, the gold oxidation behavior reinforces the picture described above suggesting that the SPR mode provides sensitive feedback regarding surface composition and charge variations, whereas the cut-off mode signal mainly reflects longer range changes in the adjacent electrolyte composition. This dual information stream enables multiscale optical electrochemical analysis, deconvoluting surface chemistry from longer-distance electrolyte while offering mechanistic insights into solid-state transformation at electrode interfaces.

### Capturing SEI formation in Li-electrolytes with additives

**Fig. 4:**
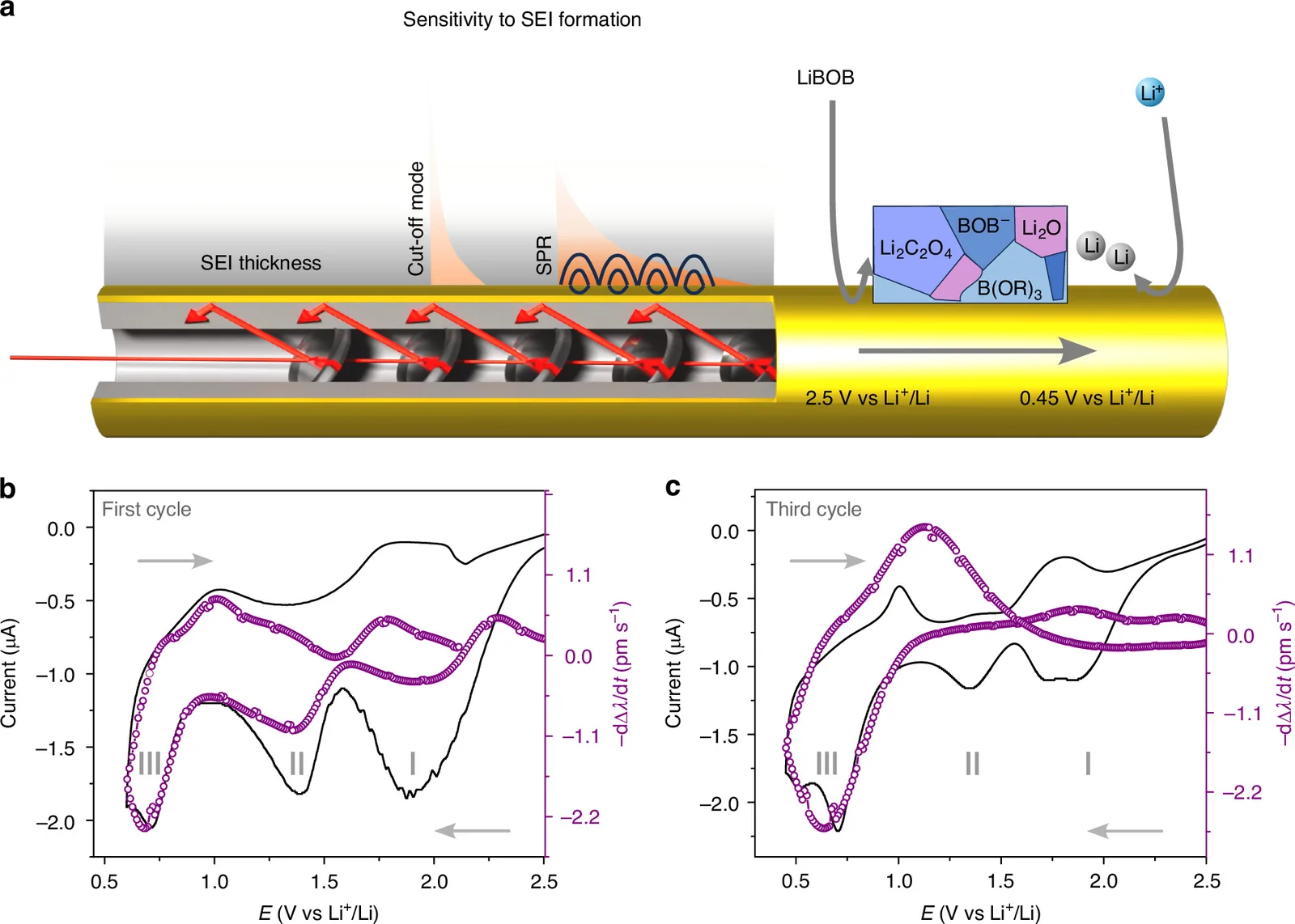
**SEI formation and Li underpotential deposition at a gold-coated TFBG fiber**. **a** Schematic of depth sensitivity compared to SEI thickness during CV of 50 mM LiBOB in 0.2 M LiClO_4_/DME; initial LiBOB reduction to organic-rich SEI with progressive inorganic enrichment and later Li underpotential deposition. **b** Selected region of first cycle CV from 2.5 V to 0.6 V vs Li^+^/Li at 2 mV s^−1^ at gold-coated TFBG fiber, using Li metal as counter and reference electrode, overlaid with the time derivative of the SPR wavelength shift ($-{{{\rm{d}}}}\Delta \lambda /{{{\rm{d}}}}t$, purple symbols). Three cathodic regimes (I–III) show optical peaks correlating with electrochemical ones. **c** Third cycle with potential window extended to 0.45 V

To extend our approach beyond aqueous environments, we investigated organic electrolytes where SEI growth and Li-alloying coexist. Measurements were performed in 0.2 M LiClO_4_ in DME with 50 mM lithium bis(oxalato)-borate (LiBOB) as additive, using Li metal as counter and reference electrode with SPR-TFBG fiber as the working electrode. LiBOB was selected because its reductive decomposition triggers a known cascade reaction driving SEI formation, schematically summarized in Fig. 4a. It is important to note here that the transition from aqueous electrolytes (RI ≈ 1.33–1.34) to organic media (RI ≈ 1.40–1.41) required modifying the TFBG sensor design, with a smaller grating tilt angle to maintain strong SPR excitation, as higher refractive index environments shift the phase-matching conditions, essential for maximizing optical response and sensitivity.

Figure 4b shows the cyclic voltammogram with three distinct cathodic activities (I–III). The first, at 1.8 V, corresponds to (per previous IR/EQCM/OEMS observations^30^) BOB anion reduction and deposition of a layer composed mainly of lithium oxalate Li_2_C_2_O_4_ and borate oxalate species. This process is observed to be accompanied by a gradual SPR signal red shift, resulting in normalized “optical mass” of 0.7 pm μC^−1^ (Fig. 4b, region I), corresponding to growth of an organic-rich layer with relatively low refractive index. Region II at ~1.3 V shows steeper optical response of 2.5 pm μC^−1^, consistent with the formation of insoluble LiOH/Li_2_O from the parasitic water reduction, significantly altering refractive index and local interfacial chemistry^31^. Lastly, reduction in region III centered around ~0.7 V induces an even larger optical shift of 3.5 pm μC^−1^, consistent with Li underpotential deposition (UPD)^32^ on Au together with accumulation of more inorganic SEI (e.g., Li_2_CO_3_ from CO_2_-derived chemistry)^30^.

Importantly, the time derivative $-{{{\rm{d}}}}\Delta \lambda /{{{\rm{d}}}}t$ (Fig. 4b) can be overlaid with electrochemical current, highlighting notable features: it exhibits peaks aligning with cathodic and anodic features in the voltammogram, confirming that SPR tracks the same surface and Faradaic processes. Notably, a small blue-shift pre-peak is resolved in ${{{\rm{d}}}}\Delta \lambda /{{{\rm{d}}}}t$ prior to region I, though less obvious in the current trace, we attribute this to early interfacial depletion preceding organic layer growth. After the first cycle, the cathodic peaks associated with LiBOB and water reduction are no longer observed in the voltammogram (Fig. S13). However, the optical response retains approximately 15% of the initial wavelength shift (regions I and II), indicating SEI growth likely continues beyond the first scan. In the third cycle, no further optical shift in this potential region is observed, consistent with the formation of fully passivating SEI both electronically and chemically (Fig. 4c). These results reveal a stage of SEI growth undetectable by electrochemistry, but captured optically and in situ with the SPR-TFBG approach. Finally, the much weaker cut-off response for these interphase-growth processes (ca. 60-fold smaller, Fig. S13b) further supports the markedly higher sensitivity of SPR to surface-confined phenomena, and therefore strengthens the interpretation proposed above for FcMeOH, where the large SPR/cut-off contrast was assigned to an interfacial adsorption/desorption contribution.

Expanding the potential window from 0.6 to 0.45 V allowed us to probe the full optical sensitivity to UPD process while avoiding Au-Li alloying at lower potentials. On the reverse scan, a Li stripping peak at ~1 V corresponds to 215 μC cm^−2^, equivalent to 1 electron per Au (111) surface atom^32^. This stripping process is characterized by a sensitivity of 14 pm μC^−1^ (Fig. S13), which compared to plating, yields optical reversibility of 68% per cycle. The missing 32% reflects continued SEI growth and partial Li trapping at Au-defect/grain boundary/low-energy sites. These findings highlight the selectivity of our method in disentangling concurrent interfacial reaction pathways.

**Fig. 5:**
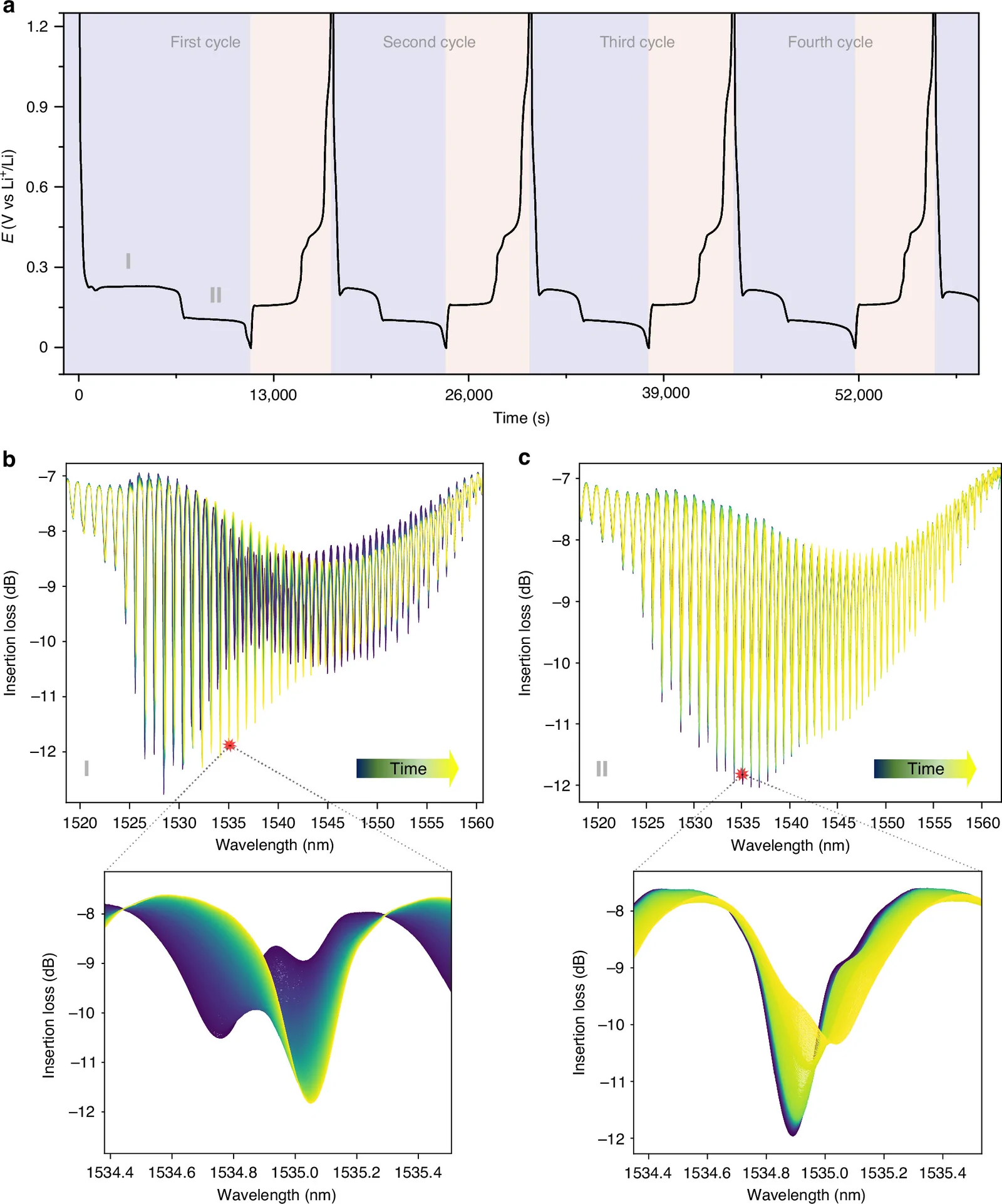
**Operando monitoring of lithiation and delithiation cycles of a gold-coated fiber**. **a** Galvanostatic lithiation (blue) and delithiation (pink) of an Au-coated fiber in 50 mM LiBOB in 0.2 M LiClO_4_/DME at 1.5 C rate. SPR-TFBG spectra through (**b**) first Au → δ-AuLi_1.6_ (I) at 0.23 V and **c** second δ-AuLi_1.6_ → AuLi_3_ (II) at 0.12 V plateaus of gold-lithium alloy formation

We next lowered the working potential to 0 V in order to trigger Li–Au alloy reactions while using galvanostatic control with a discharge current of 1.25 μA. We then clearly observed two well-known Li-induced reduction plateaus occurring at 0.23 and 0.12 V, corresponding respectively to the Au → δ-AuLi_1.6_ (I) and δ-AuLi_1.6_ → AuLi_3_ (II) alloy reactions (Fig. 5a). Upon entering the first alloying plateau, the SPR-TFBG spectrum undergoes a pronounced change (Fig. 5b), with a deeper, broader resonance dip red-shifted over 10 nm. This spectral evolution, consistent with changes during cyclic voltammetry (Fig. S14), reflects an abrupt change in the film’s dielectric function as Li incorporated into Au. In the second plateau the resonance continues red shifting, but amplitude changes dominate (Fig. 5b). We attribute this to microstructural damage during deep lithiation^33^, crack formation and incipient porosity partitioning the film into nano-islands, introducing elastic scattering and resonance dampening. Consistent with this interpretation, we observe concomitant changes in fiber Bragg resonance (Fig. S14d) and post-mortem SEM evidence of cracking/porosity in the Au coating (Fig. S15). Such drastic irreversible modifications induce profound spectral distortions, underscoring limitations of tracking a single resonance minimum, but also establish a clear boundary, marking the onset of completely different chemical and morphological environments for the SPR active material, of critical importance for identifying possible (or likely) irreversible reactions.

To overcome this, we implemented an alternative approach of monitoring spectral envelope area rather than narrow resonance region. As shown in Fig. S16a-c, after the initial SPR transformation during the first cycle, subsequent lithiation-delithiation cycles can be reliably tracked by variations in the envelope amplitude. Importantly, the method reveals a progressive loss of delithiation reversibility of ~10% per cycle, highlighting the sensitivity of our optical readout to cumulative irreversible structural changes.

### Optical monitoring of electrodeposition and conversion in Prussian blue films

**Fig. 6:**
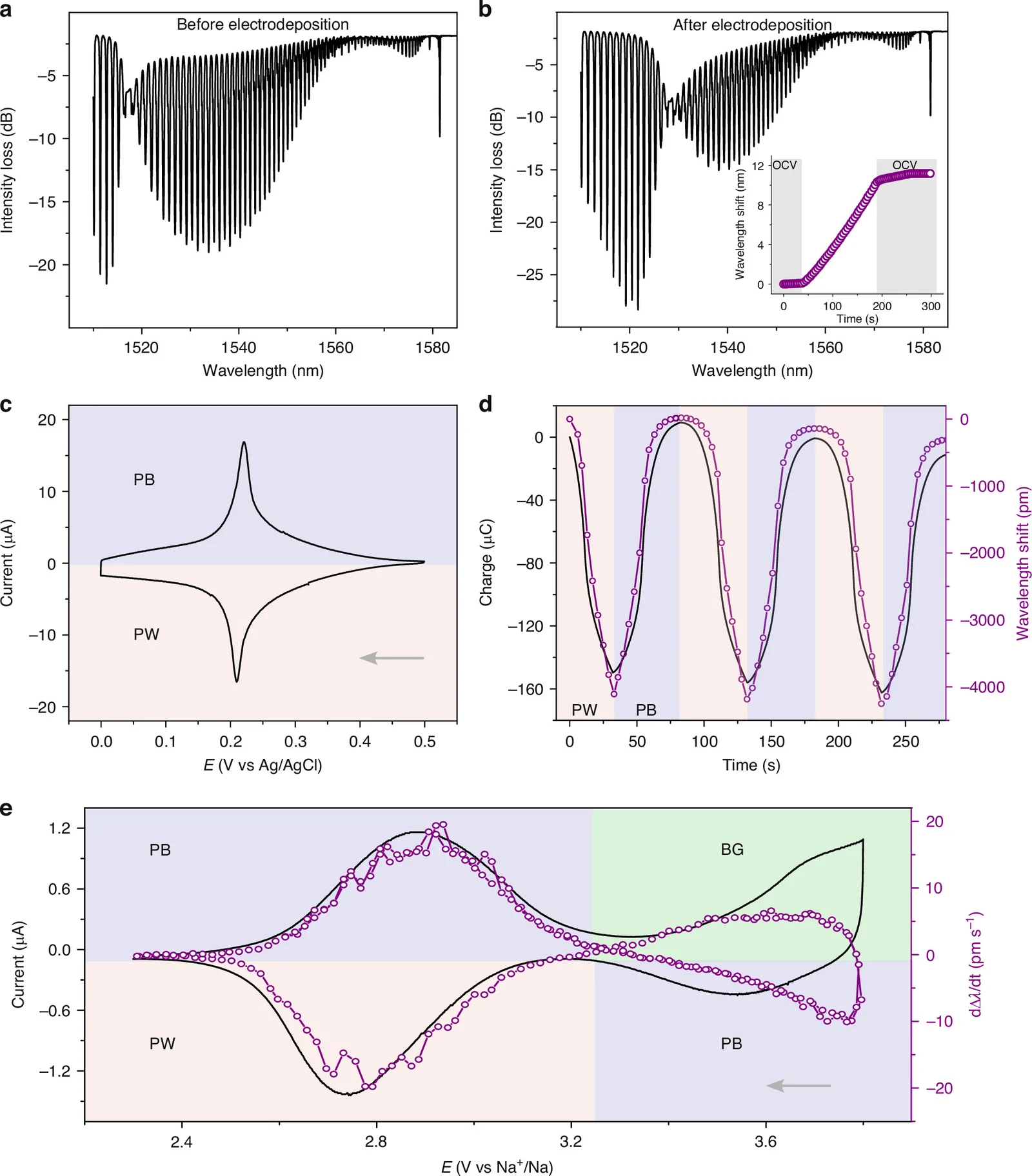
**Optical monitoring of electrodeposition and conversion in Prussian blue films**. **a** Measured SPR-TFBG transmission spectrum before and **b** after electrodeposition of PB via chronopotentiometry at -7 μA cm^−2^ in electrolytic solution. **c** Cyclic voltammograms of electrodeposited Prussian blue phase on SPR-TFBG in 0.25 M K_2_SO_4_ pH 3.5 at 10 mV s^−1^. **d** SPR response to reversible K⁺ insertion and extraction over three cycles. **e** CV of activated PB in 0.7 M NaPF_6_ in propylene carbonate (PC) at 5 mV s^−1^ using sodium metal as both counter and reference electrode, overlaid with the time derivative of the SPR wavelength shift (${{{\rm{d}}}}\Delta \lambda /{{{\rm{d}}}}t$, purple symbols) measured during electrochemical monitoring of Na⁺ insertion/extraction

To demonstrate the full potential of multimodal SPR-TFBG sensors as working electrodes, our study extends further to monitoring electrodeposition and alkali uptake processes of Prussian blue (PB) Fe_4_^III^[Fe^II^(CN)_6_]_3,_ a widely used insertion compound in aqueous and non-aqueous Li(Na)K-ion batteries. A gold-coated TFBG fiber served as working electrode in a three-electrode system with platinum counter and Ag/AgCl reference electrodes immersed in acidic solution containing ferric and ferricyanide ions (see “Methods” for details)^34^. The cathodic electrodeposition process for synthesizing PB was undertaken with a constant current density of −7 μA cm^−2^, while simultaneously recording the optical signal (Fig. 6a, b). The corresponding SPR signal displayed a linear (with time/charge) red shift up to 10.7 nm (Fig. 6b, inset), attributed to continuous PB film growth on gold. This shift is consistent with constant current and replacement of the surrounding 2 mM electrolyte (*n* ≈ 1.3326) with higher index PB layer (*n* ≈ 1.5808)^35^. Parallel electrodeposition performed on gold-coated glass slides under identical conditions revealed, as deduced from X-ray diffraction, the formation of the cubic phase Fe_4_^III^[Fe^II^(CN)_6_]_3_ (Fig. S17) with an a-axis of 10.124(8) Å.

The electrodeposited PB film, now with a Au “current-collector”, serves as a full electrode which could then be cycled at 10 mV s^−1^ in 0.25 M K_2_SO_4_ at pH 3.5 to induce K^+^ insertion and partial Fe leaching (Fig. 6c), as currently done in the literature^36^. From the cumulated charge during reversible PB/PW conversion, we estimated the film thickness to be 11.6 nm (assuming 100% Faradaic efficiency, see Supplementary Information). Since the PB film was grown under constant-current protocol, thickness should increase linearly with time. Consistent with this, the observed SPR red shift with electrodeposition time (inset, Fig. 6b) indicates strong linear correlation between SPR wavelength shift $\Delta \lambda$ and film thickness: ${t}_{{{{\rm{film}}}}}=1.08\,\Delta \lambda$. This proportionality supports using SPR-TFBG sensors for real-time optical tracking of nanoscale film growth during electrodeposition.

Figure 6d shows that SPR-TFBG nicely captures the reversible Prussian blue/Prussian white (PB/PW) phase transition during cyclic voltammetry, with reversible blue and red shifts of approximately 4 nm attributed to uptake (reduction to PW) and removal (oxidation to PB) of K^+^-ion, respectively. Notably, the optical response cannot be explained solely by refractive index changes and also reflects absorption variations: PB exhibits broad absorption at ~700 nm extending to near-IR^37^, while PW is optically transparent at relevant wavelengths. Therefore, the blue shift upon PB reduction is attributed to a decrease in the imaginary part of the effective refractive index^38^, consistent with prior opto-electrochemical studies on electrochromic materials including PB^39,40^.

The PB/PW transition kinetics were analyzed by fitting SPR shifts with sigmoidal functions, capturing characteristic features of ion-driven optical changes during cycling (Fig. S18) with a sensitivity of 30 pm μC^−1^. In the first cycle, slight asymmetry was observed between insertion and extraction, with faster transition during K^+^ extraction (10.5% smaller transition time, Fig. S18d), likely due to fine structural rearrangements during first ion exchange. This asymmetry progressively diminishes over repeated cycles, with transition time difference reducing to 4% by the third cycle, while electrochemical response asymmetry also decreasing from 15% to 8%. These results demonstrate SPR-TFBG’s ability to monitor solid-state transformations in PB films in real time, capturing both refractive and absorptive changes, reveal their potential for probing intercalation reaction in electrochemical storage materials. As for LiBOB-derived interphase formation and FcMeOH oxidation, the much smaller cut-off response (Fig. S19) confirms that the large SPR signal is here again dominated by a thin interfacial layer process.

Extending the applicability toward realistic battery environments, we investigated Na^+^ insertion reversibility into PB films in organic electrolyte. Figure 6e shows cyclic voltammetry of KFe^III^[Fe^II^(CN)_6_] films in 0.7 M NaPF_6_ in propylene carbonate, revealing reversible PB/PW transformation marked by characteristic redox peak pair. Expanding the potential window beyond 3.5 V vs Na⁺/Na resulted in further oxidation to Berlin Green (BG, Fe^III^[Fe^III^(CN)_6_]) with extra Na^+^ removal. The temporal evolution of the wavelength shift together with corresponding ${{{\rm{d}}}}\Delta \lambda /{{{\rm{d}}}}t$ profile reveals the characteristic kinetics of the associated phase transitions. As before, charge and SPR shifts were fitted by sigmoidal transitions for both Na⁺ insertion and extraction (Fig. S20a-d). The first cycle exhibited an asymmetry opposite to aqueous electrolyte: the SPR kinetics during Na⁺ insertion (charge) were 10.6% faster than extraction (discharge), mirroring electrochemical charging (9.8% faster). In the second cycle, this asymmetry reversed: Na^+^ insertion became 5.1% slower than extraction, again mirroring electrochemical response (4.4% slower). This inversion in kinetics consists of early-cycle structural reorganization altering Na⁺ insertion pathways over time. The fact that this translates in both charge and optical measurement suggests it relates to structural reorganization rather than solely ion transport mechanisms and underscores the utility of novel methods like SPR-TFBG sensing in paving new pathways to more holistic understandings of complex materials and interfaces.

### Toward practical deployment

**Fig. 7:**
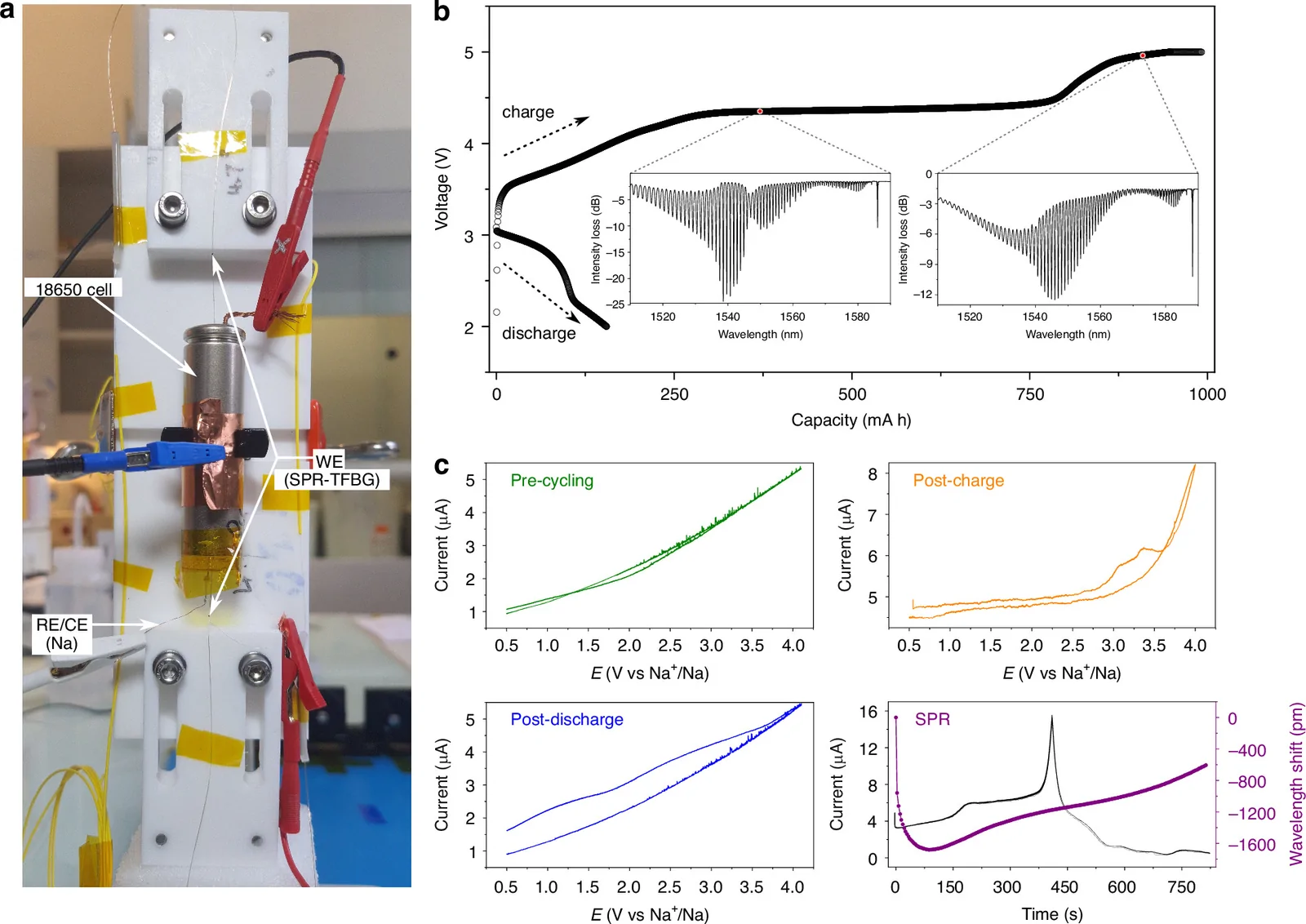
**Operando sensing inside a sealed 18650 Na-ion cell**. **a** Cell assembly (NVPF/HC, 1 M NaPF_6_ in DMC) with an SPR-TFBG fiber integrated at the center of the jelly roll. The fiber acts as an embedded optical/electrochemical sensing electrode (denoted working electrode), while a sodium-metal is used as a reference/counter electrode for this electrochemical operando sensing inside the sealed cell. **b** Galvanostatic charge-discharge profile of the NVPF/HC full cell (2–5 V at C/5) with corresponding SPR-TFBG transmission spectra. The voltage axis refers to the full-cell voltage. **c** In situ diagnostic cyclic voltammograms recorded locally at the embedded fiber sensing electrode against Na^+^/Na at successive formation stages (pre-cycling, post-charge, post-discharge); the bottom right panel shows simultaneous SPR wavelength shift and current recorded during one diagnostic polarization (from 0 to 4 V vs Na^+^/Na at scan rate 1 mV s^−1^)

The demonstrations above establish, in open three-electrode configurations, that the gold-coated SPR-TFBG can resolve and quantify interfacial processes spanning double-layer charging, Faradaic redox, interphase formation and ion intercalation. A decisive question for practical deployment is whether this capability survives translation into a realistic, sealed cell format, where the fiber must operate within a confined, opaque and chemically aggressive environment rather than an open beaker. To address this, we embedded an SPR-TFBG sensor within a commercial-format 18650 cylindrical Na-ion cell made of a sodium vanadium fluorophosphate (NVPF) as the positive electrode and hard carbon (HC), as the negative electrode with 1 M NaPF_6_ in dimethylcarbonate (DMC) as the electrolyte (NVPF/HC, 1 M NaPF_6_ in DMC). The fiber was positioned at the center of the jelly roll so that it samples the interior of the wound electrode assembly (Fig. 7a). In this configuration, the fiber is not one of the battery electrodes but an embedded sensor serving simultaneously as the optical probe and as an electroactive sensing electrode. As a sensing electrode, the gold coating of the fiber is polarized against a sodium-metal reference/counter electrode introduced to enable operando interrogation inside the sealed cell and combining the optical and electrochemical functionalities developed throughout this work.

Galvanostatic charge-discharge of the NVPF/HC full cell between 2.0 and 5.0 V at C/5 was recorded while continuously acquiring SPR-TFBG transmission spectra (Fig. 7b). The deliberately extended upper cut-off, beyond the nominal NVPF redox window, was chosen to drive the cell into the high-voltage regime where parasitic processes become significant. The plasmonic signature (two example spectra given in Fig. 7b) tracked the evolving interior environment throughout cycling, with a pronounced loss of the SPR resonance dip as the cell voltage exceeded 4.5 V. This optical change indicates that the local or interfacial refractive index and absorption at the embedded fiber surface is modified during high-voltage cell operation.

Electroactive species generated at the cell electrodes migrate toward the fiber and modify its surface environment, which can be probed both electrochemically and optically. Diagnostic cyclic voltammograms were recorded at the embedded fiber sensing electrode, against the sodium reference/counter electrode, at successive stages of the formation sequence, pre-cycling, post-charge, and post-discharge (Fig. 7c). Before cycling, the featureless current response monotonically increasing with potential, reflects the ionic conductivity background of the fresh electrolyte with no resolved redox active species.

After charging the full NVPF/HC cell to 5 V, beyond the nominal NVPF redox window and into the regime where carbonate-based electrolytes are known to undergo anodic decomposition, the fiber CV develops new anodic features above ~3 V vs Na^+^/Na ^41^. As the Au fiber electrode is chemically inert over this window, these features are not intrinsic to the sensor and are attributed to electroactive species generated by high-voltage electrolyte oxidation at the cell electrodes and detected locally at the fiber. Their absence of corresponding cathodic features indicates electrochemically irreversible oxidation. Following discharge, the CV largely recovers its initial featureless profile, consistent with conversion of these species into electrochemically inactive products, such as insoluble decomposition compounds that precipitate from the electrolyte and are thereby excluded from the probed volume. A residual baseline shift persists, however, indicating incomplete restoration of the local electrolyte environment.

The bottom right panel of Fig. 7c shows the SPR wavelength shift and current recorded simultaneously during a diagnostic polarization of the fiber sensing opto-electrode when the charged cell shows a cell voltage of 4.5 V (400 mA h). The corresponding CV is intermediate between the pre-cycling and post-charge indicating the progressive transformation of the cell electrolyte. The evolution of the SPR wavelength shift during this sweep recorded inside the sealed cell, shows a sharp blue shift at the start followed by a red shift in the region of the oxidation peak at ca. 180 s (ca. 3 V vs Na^+^/Na). The complete deciphering of the electrolyte composition and correlated opto-electrochemical response of the sensor is beyond the scope of this work, however this clearly shows that the sensor reports on the evolving state of the buried electrode rather than on a static environment.

Taken together, these results confirm that the methodology developed in open-cell configurations translates to a sealed, commercially relevant 18650 architecture, where the embedded fiber enables operando detection of parasitic redox processes during formation through simultaneous electrochemical and optical interrogation. We present this as a feasibility demonstration; a comprehensive investigation of cell-integration strategies, together with a detailed mechanistic analysis of the high-voltage parasitic chemistry resolved here, will be the subject of a dedicated future study.

## Discussion

We have demonstrated an electroactive fiber-optic platform where a gold-coated SPR-TFBG simultaneously functions as a working electrode and optical probe. This integration enables charge-resolved tracking of interfacial electrochemical dynamics via the Δλ_SPR_-ΔQ relationship (‘optical voltammetry’), extracting mechanistic insights inaccessible with electrochemistry or optics alone. The interpretation of experimental data is grounded in a quantitative framework combining Poisson-Nernst-Planck transport, potential-dependent adsorption/desorption of ionic and molecular species, and refractometric relationships linking measured SPR wavelength shifts to local changes in bulk refractive index, charge density and surface coverage.

This platform uniquely resolves three classes of interfacial phenomena: double-layer dynamics, Faradaic reactions, and solid-state transformations spanning nanoscale surface and micrometer-scale bulk length scales. We demonstrated this capability in aqueous systems through Au electrode restructuring, adsorption/desorption dynamics during ferrocenemethanol redox and Au oxide formation in sulfuric acid. Extension to organic electrolytes bridged a critical gap for battery-relevant diagnostics, enabling tracking of native SEI formation on Au with unprecedented interfacial chemical specificity. The platform’s sensitivity to electrodeposition processes was further validated through Prussian blue nucleation, thin-film growth and ion intercalation dynamics, achieving sub-monolayer optical resolution. These diverse experimental validations underscore the versatility of the SPR-TFBG architecture for mechanistic interrogation across electrochemical interfaces.

Critically, we translated this platform into a realistic battery architecture by embedding the SPR-TFBG within a sealed cylindrical 18650 Na-ion cell (NVPF/HC, 1 M NaPF_6_ in DMC). In this operando configuration, the fiber electrode enabled real-time optical voltammetry detection of parasitic redox processes during the cell formation cycle. This sealed-cell validation demonstrates that our methodology successfully bridges laboratory-scale electrochemical characterization and practical battery diagnostics, a prerequisite for deployment in industrial battery development pipelines. Comprehensive investigation of cell integration strategies and detailed mechanistic analysis in such practical formats will be the subject of future work.

Current limitations center on fiber coating reproducibility: precise control of Au density and thickness on cylindrical surfaces remains technically challenging due to adhesion and conformality constraints. To ensure consistency, all presented results were reproduced independently at least twice. Standardization protocols and advanced deposition techniques, including atomic layer deposition (ALD) and electrochemical plating, offer promising pathways to address this challenge in future development. Additionally, advances in optical-electrochemical interfacing are needed to enable more user-friendly operation and broader adoption. We have prepared detailed technical guidance in the Supplementary Information section *“Practical guidance towards SPR-TFBG sensing”* which addresses modal sensitivities, probing depths, and quantitative interpretations, providing a resource for researchers pursuing similar approaches.

The innovations established here, real-time spectral demodulation via Fourier transform analysis, depth-resolved optical sensing, and active electrochemical probing, create opportunities for advancing in situ monitoring across energy storage systems. Future work will leverage advanced data analytics and machine learning to extract real-time electrode aging signatures, quantify catalyst stability degradation, and identify performance-limiting mechanisms. Extending this optical-electrochemical platform to Li-ion, Na-ion, all-solid-state and flow-battery systems will establish operando diagnostics as a standard characterization tool for battery development and quality assurance at scale. Such integration could accelerate the optimization of electrolyte formulations, interphase coatings and electrode microstructures critical for next-generation energy storage.

This work establishes operando optical-electrochemical diagnostics as a rigorous platform for mechanistic interrogation of battery interfaces, bridging the gap between laboratory characterization and industrial-scale diagnostics. The ability of this platform to resolve sub-monolayer interfacial phenomena and bulk electrolyte dynamics simultaneously in sealed cells redefines how we observe and optimize ion transport, interphase chemistry and electrolyte performance, capabilities essential for accelerating next-generation battery development and advancing the energy storage technologies critical to climate mitigation and global energy security.

## Materials and methods

### TFBG sensor fabrication

The fabrication process of SPR-TFBGs involves several steps: A silica telecommunication-grade optical fiber (Corning SMF-28) is first hydrogen loaded at ~200 bar and 60 °C for 30 h to enhance its photosensitivity. Then, using a phase mask technique, a tilted Bragg grating is inscribed within the fiber core. An excimer laser-based system (Noria, from Northlab Photonics) operating at 193 nm is used in conjunction with an 8° and 6.4° tilted phase mask with a period of 1100 nm. To eliminate hydrogen in excess, the fiber is heated at 100 °C for 24 h. Finally, a 50 nm-thick gold layer is deposited on the fiber using a Spuco sputter-coater equipped with 2 in. magnetron modules operating at a 250W-RF power supply. The deposition process is monitored using an in-built quartz microbalance with a resolution of 0.1 nm.

### Operando measurement by TFBG sensors

To achieve TFBG sensor operando measurement, the optical transmission spectra were recorded (5 s/spectra) by an optical interrogator (CTP10 and T100S-HP, EXFO SOLUTIONS) with a resolution of 1 pm for wavelength ranging from 1510 to 1600 nm.

### Electrochemical measurements

The electrochemical measurements were performed on MPG2 or SP-200 potentiostat (Biologic, France) at 25 °C inside a temperature-controlled oven (Memmert, ±0.1 °C) for aqueous measurements. The electroactive part of the Au-coated fiber in contact with the electrolyte was $L=3.7\,{{{\rm{cm}}}}$ long, giving a nominal geometric cylindrical area of the immersed for the $d=125\,{{{\rm{\mu }}}}{{{\rm{m}}}}$ diameter Au-coated fiber, of ${A}_{{{\mathrm{geom}}}}=\pi {dL}=0.145\,{{{\mathrm{cm}}}}^{2}$. This area, not corrected for Au roughness or local coating non-uniformity, is used for current density and Prussian blue thickness evaluation. For aqueous electrolytes, a platinum (Pt) wire (Alfa Aesar) was the counter electrode and Ag/AgCl (3 M NaCl) (Biologic) served as the reference electrode, and the 50 nm gold-coated TFBG as the working/sensing electrode. Electrolyte K_2_SO_4_ (99% Sigma-Aldrich) was diluted to correct concentration with Milli-Q water (Q-POD Merck) and used as a base for dissolution of Ferrocenemethanol (97% Sigma-Aldrich) to 5 mM concentration. Concentrated H_2_SO_4_ (98% Sigma-Aldrich) was diluted with Milli-Q to 0.5 M concentration.

For measurement in non-aqueous electrolytes (dried 24 h over using molecular sieves) all the cell parts were dried overnight at 80 °C before being assembled and measured in an Ar-filled glovebox (MBraun, O_2_ and H_2_O < 0.1 ppm). 50 nm gold-coated TFBG working electrode with lithium and sodium metal (99.9%, Sigma-Aldrich) of 8 mm were punched to be used as both counter and reference electrodes. 1,2-dimethoxyethane (DME) (Sigma-Aldrich, 99.9%, inhibitor-free) was dried over molecular sieves (H_2_O content <10 ppm) and mixed to 0.2 M with LiClO_4_ (99.99% trace metal basis, battery-grade, Sigma-Aldrich). Lithium bis(oxalato)borate, LiBOB (Sigma Aldrich), was added to the base electrolyte to achieve the correct concentration (50 mM LiBOB). Commercial-format 18650 cylindrical Na-ion (TIAMAT) cell made of a sodium vanadium fluorophosphate (NVPF) as the positive electrode and hard carbon (HC), as the negative electrode with 1 M NaPF_6_ in dimethylcarbonate (DMC) as the electrolyte (NVPF/HC, 1 M NaPF_6_ in DMC).

### Electrodeposition and insertion study

A fresh precursor solution of 2 mM K_3_Fe(CN)_6_ (99% Sigma-Aldrich) and 2 mM FeCl_3_x6H_2_O (99% Sigma-Aldrich) in 0.01 M HCl (37% Sigma-Aldrich) was prepared for PB electrodeposition using chronoamperometry at -7 μA cm^−2^ (1 μA for 0.145 cm^2^ surface area). Our modified electrochemical cell consisted of 50 nm gold coated TFBG working electrode, platinum (Pt) wire as a counter electrode and Ag/AgCl (3 M NaCl) reference electrode. The electrochemical activation of electrodeposited PB was performed in 0.25 M K_2_SO_4_ (99% Sigma-Aldrich) solution acidified to pH 3.5 with H_2_SO_4_ in the potential window from −0.2 V to 1.2 V. After this step, the films can be cycled in the same aqueous electrolyte or in a non-aqueous electrolyte 0.7 M NaPF_6_ (Stella Chemica) or 0.25 M KPF_6_ (99% Sigma-Aldrich) in PC (E-Lyte). For the latter, the films were dried overnight at 30 °C.

### Materials characterization

X-ray diffraction (XRD) was performed to confirm the success of the synthesis using a BRUKER D8 Advance diffractometer with Cu Kα radiation (*λKα*1 = 1.54056 Å, *λKα*2 = 1.54439 Å). Rietveld refinement was performed to obtain structural parameters as implemented in the FullProf suite. The crystal structures shown in this work were drawn with the VESTA visualization program using the crystallographic information obtained from Rietveld refinements.

Scanning Electron Microscopy (SEM) was conducted with a SU-70 Hitachi FEG-SEM (typically with an accelerating voltage of 15 keV).

### COMSOL modeling

The DLC and redox probe diffusion experiments were reproduced by implementing a COMSOL model taking account of the tertiary current distribution using the electrostatic module, and diffusion of diluted species module for both the electrolyte and the redox probe, and considering surface adsorption/desorption dynamics, solving ordinary differential equation modules at the electrode surface. The cut-off and SPR wavelength shifts are evaluated considering Eqs. 1–5 mentioned above. The model developed in COMSOL v5.5 in 1D geometry is detailed in SI.

## Supplementary information

Supplementary Information for: Electroactive fiber optics for real-time, multiscale probing of ion transport and interfacial chemistry

## Data Availability

The datasets generated and analyzed during the current study are available from the corresponding author upon reasonable request.
