# Supplementary material for: Electroactive fiber optics for real-time, multiscale probing of ion transport and interfacial chemistry
Source: Light Sci Appl. 2026 Nov 9;15:399. doi: 10.1038/s41377-026-02521-0 (PMC13748620; doi:10.1038/s41377-026-02521-0)
Supplement: Supplementary file 1 [file 41377_2026_2521_MOESM1_ESM.docx]

**Supplementary Information for:**

**Electroactive fiber optics for real-time, multiscale probing of ion transport and interfacial chemistry**

Tatjana Šafarik^1^, Frédéric Kanoufi^2,3^, Médéric Loyez^4^, Hadrien Fasseaux^4^, David Aymé-Perrot^5^, Nilanka M. Keppetipola^1^, Murugasenapathi N K^1^, Steven T. Boles^6^, Christophe Caucheteur^4^, Jean-Marie Tarascon^1*^

1 Chimie du Solide et de l’Energie, UMR 8260 CNRS, Collège de France, 75005 Paris, France

Email: [jean-marie.tarascon@college-de-france.fr](mailto:jean-marie.tarascon@college-de-france.fr)

2 Université Paris Cité, CNRS, ITODYS, F-75013 Paris, France

3 Laboratoire de Physique de l’École normale supérieure, ENS, Université PSL, CNRS, Sorbonne Université, Université Paris Cité, F-75005 Paris, France

4 Electromagnetism and Telecommunication Department, Advanced Photonic Sensors Unit, University of Mons (UMONS), 7000 Mons, Belgium

5 TotalEnergies SE, OneTech, 2 Place Jean Millier, La Défense 6, 92078 Paris, France

6 Department of Energy and Process Engineering, Faculty of Engineering, Norwegian University of Science and Technology (NTNU), 7491 Trondheim, Norway

Tilted fiber Bragg gratings (TFBGs) are permanent periodic modulations of the refractive index inscribed within the core of an optical fiber, with the grating plane intentionally tilted relative to the fiber axis. This tilt enables coupling of core-guided light into a broad set of cladding modes, where the resulting evanescent fields extend beyond the fiber boundary and interact sensitively with the external environment. This mechanism forms the basis for highly sensitive refractometric sensing. In addition to cladding mode coupling, TFBGs also maintain a core-guided Bragg resonance, which remains unaffected by the surrounding medium and instead responds to axial strain and temperature changes along the fiber. Each cladding mode is associated with a distinct effective refractive index, and thus with a unique spectral dip in the transmission spectrum over a typical range of 1510 - 1560 nm. These dips shift as the optical properties of the surrounding medium change. In particular, shifting occurs towards longer wavelengths (so-called red shift) as the surrounding refractive index (SRI) increases. The highest refractometric sensitivity is achieved near the cut-off wavelength marked as transition between cladding and radiative modes, where the effective refractive index matches that of the medium. Additionally, amplitude shifting indicate changes in the intensity of light coupled between the core and cladding modes. These shifts are dependent on the distribution and polarization of the electromagnetic field of the mode across the fiber cross-section. As the SRI increases, the cladding mode becomes less confined, resulting in enhanced leakage of the electromagnetic field into the surrounding medium and a corresponding decrease in resonance amplitude (in dB). Throughout this study, resonance wavelength shifts were selected as the primary metric to track SPR and cut-off mode responses, owing to their robustness against polarization fluctuations and experimental instabilities. While amplitude variations are more frequently reported in the literature, they are more prone to artifacts and thus less suitable for quantitative comparisons. To acquire spectral information, TFBGs are typically interrogated in transmission, positioned between broadband optical light source (in the near-infrared wavelength range) and optical spectrum analyzer (Fig. 1). A 50 nm thick gold coating of TFBG enhances the overall sensitivity to local changes in refractive index (RI) by approximately ten times, attributed to surface plasmons excited by evanescent electromagnetic fields at the nanoscale. Plasmon excitation at the gold surface leads to selective attenuation of specific cladding modes, manifested as sharp dips in the transmission spectrum, indicated as SPR in Fig. 1.

Typically, P-polarized light relative to the tilt plane of the grating is necessary to match with plasmonic resonance condition, as S-polarized light cannot excite surface plasmons because its electric field is entirely parallel to the interface, lacking the normal (orthogonal) component required to induce charge separation and drive plasmon oscillations. Accordingly, a polarization controller and polarizer were employed to ensure SPR excitation. The precise position and intensity of the SPR response are influenced by the gold film properties, tilt angle, incident polarization, and surrounding refractive index.

**Fig. S1. a** Single-peak tracking method and **b** demodulation Fourier transform analysis performed on the same experimental data set.

**Fig. S2. a** Transmission spectra of SPR-TFBG in 0.1 M, 0.25 M and 0.5 M K_2_SO_4_ solutions, highlighting the SPR region (purple box) and cut-off mode (red asterisk). **b** SPR and cut-off wavelength shifts for fibers placed in bulk aqueous K_2_SO_4_ (0.1 M - 0.65 M) solutions. **c** SPR sensitivity calibration as a function of the solution refractive index.

**Fig. S3.** **a** Capacitive current measured while varying the scan rate from 5 mV s^-1^ (dark blue) to 35 mV s^-1^ (light blue), cycled from OCV to OCV and **b** corresponding SPR shift. **c** Capacitive current measured while changing scan rate from 5 mV s^-1^ (dark blue) to 35 mV s^-1^ (light blue) with each cycle starting at OCV and ending at 0 V, with five-minute rest period between successive scan rate changes and **d** the corresponding SPR shift.

**Fig. S4. a** Accumulated charge and **b** SPR wavelength shift as a function of scan rate. **c** Their linear correlation demonstrates that the optical response scales with the accumulated interfacial charge.

**Fig. S5.** **a** Correlation between the SPR wavelength shift and accumulated charge during second electrochemical cycle in potential window from 0 V to 0.5 V at 5 mV s^-1^ in 0.1 M K_2_SO_4_. **b** Accumulated charge plotted against SPR wavelength shift. **c** Linear fit yielding a slope of 4.75 pm μC^-1^ and an R² value of 0.9928.

**Fig. S6. a** SPR wavelength shift (purple) and charge (black) response of the gold-coated SPR-TFBG electrode during sequential potential steps (0 V, 0.15 V, 0.35 V, and 0.5 V vs Ag/AgCl) in 0.1 M K_2_SO_4_, each applied over two minute period and followed by a five minute open-circuit relaxation period. **b** Wavelength shifts plotted as function of time for each OCV region.

**Fig. S7. a** Integrated SPR wavelength shift during polarization at 0 V in 0.1 M K_2_SO_4_. **b** Integrated accumulated charge during the same polarization period. **c** Integrated SPR wavelength shift during the subsequent relaxation period.

**Comsol Model for double-layer charging and FcMeOH electrochemistry**

All simulations were performed in COMSOL Multiphysics (version 5.5) using a **1D geometry** normal to the Au/electrolyte interface. The spatial domain extends from the electrode surface (*z* = 0) to the bulk electrolyte (*z* = *L*, typically 2 mm), where concentrations and potential are fixed at their bulk values.

- **Physics and species**

Coupled **Electrostatics (ES)** and **Transport of Diluted Species (TDS)** interfaces were used to solve a Poisson–Nernst–Planck–type description for all mobile species:

- Supporting electrolyte ions: K^+^, SO_4_^2-^
- Redox couple: FcMeOH (Fc) and FcMeOH⁺ (Fc⁺).

The electrostatic potential *ϕ*(*z*,*t*) satisfies Poisson’s equation with the local charge density given by the sum of ionic concentrations. Species transport is described by diffusion and migration in the electric field. Diffusion coefficients are taken as 2$\times$10^-5^ and 7$\times$10^-6^ cm^2^ s^-1^ respectively for K^+^, SO_4_^2-^ and the FcMeOH species and bulk concentrations as their experimental values.

- **Boundary conditions and electrochemical driving**

At the **electrode boundary** (*z* = 0), the potential is imposed as a time-dependent function *V*_app_(*t*) corresponding either to:

- cyclic voltammetry (triangular waveform defined via a periodic function of time), or
- chronoamperometry (potentiostatic steps followed by open-circuit relaxation).

A Stern-like boundary condition was used to relate the electrode potential, the potential drop in the compact layer, and the solution potential at *z* = 0. At the **bulk boundary** (*z* = *L*), the potential is grounded (*ϕ* = 0) and all species concentrations are fixed to their bulk values, enforcing a well-stirred reservoir.

For Fc/Fc⁺, a Butler-Volmer-type flux condition at *z* = 0 describes the interfacial electron transfer, with rates chosen such that the simulated voltammograms reproduce the experimental peak currents and *E*₁_/_₂ values.

- **Surface adsorption / desorption**

Specific adsorption of sulphate and Fc species is represented by **boundary ordinary differential equations (ODEs)** for the surface coverages *θ*_SO4_, *θ*_Fc_, *θ*_Fc⁺_ at *z* = 0:

$\frac{d\theta_{i}}{dt}=k_{\mathrm{ads},i}\left( E \right)c_{i}\left( 0,t \right)\left( 1-\theta_{i} \right)-k_{des,i}(E)\theta_{i}$	(SI1)

where *i* denotes SO_4_^2-^ or Fc/Fc⁺, and *c_i_*(0,*t*) is the interfacial concentration. The rate constants are taken as simple exponential functions of potential relative to the point of zero charge, pzc:

$k_{ads,i}\left( E \right)= k_{\mathrm{ads},i,\mathrm{pzc}}exp\left( \alpha_{i}\beta_{i}\frac{F}{RT}(E-E_{\mathrm{pzc}}) \right)$	(SI2)

$k_{\mathrm{des},i}\left( E \right)= k_{\mathrm{des},i,\mathrm{pzc}}exp\left( {-(1-\alpha}_{i})\beta_{i}\frac{F}{RT}(E-E_{\mathrm{pzc}}) \right)$	(SI3)

With $k_{i,\mathrm{pzc}}$ correspond to the rates of adsorption or desorption of species at pzc, $\alpha_{i}$ the symmetry factor and $\beta_{i}$ an additional term used for ionic species to account for their effective charge in its adsorbed state. Typically $\alpha_{i}=0.5$ and $\beta_{i}=0.4$

The values of the adsorption/desorption rate constants at pzc are estimated using characteristic time $\tau_{\mathrm{app}}$ for the overall adsorption process (in the 10^2^-10^3^ s range).

$k_{\mathrm{ads},i,\mathrm{pzc}}= \frac{\theta_{i,\mathrm{pzc}}\Gamma_{\max}}{\tau_{\mathrm{app}}C_{i}^{\mathrm{bulk}}}$	(SI4)

$k_{\mathrm{des},i,\mathrm{pzc}}= \frac{{1-\theta}_{i,\mathrm{pzc}}}{\tau_{\mathrm{app}}}$	(SI5)

With $\Gamma_{\max}$the maximum surface coverage and $C_{i}^{\mathrm{bulk}}$ the bulk concentration of the adsorbing species.

They were adjusted to match the experimentally observed time scale (tens of seconds) and amplitudes of the slow SPR shifts during polarization and OCV (Figures 2d, and S8a). The adsorbed species contribute to the total surface charge density and to the optical response via their effective polarizability.

- **Solution strategy**

Each simulation consist of:

1. A **stationary step** to compute the equilibrium potential and concentration profiles at the initial potential (typically near the pzc) including steady-state adsorption coverage.
2. A **time-dependent step** where *V*_app_(*t*), species concentrations, potential, and coverages evolve according to the ES, TDS and ODE equations.

This two-step procedure ensures realistic initial conditions for the transient response to potential sweeps or steps.

- **Optical response (SPR and cut-off modes)**

The local refractive index *n*(*z*,*t*) is computed from the simulated concentration profiles assuming that the refractive index of a solution is an additive function of its components and changes linearly with the different solute concentrations^[1]^:

$n\left( z,t \right)=n_{\mathrm{solvent}}+\sum_{i} \left( \frac{dn}{dc} \right)_{i}c_{i}(z,t)$	(SI6)

with $\left( \frac{dn}{dc} \right)_{i}$ the RI change with concentration and $c_{i}(z,t)$ the local concentration of species *i*. The variation in refractive index, $\Delta n\left( z,t \right)$ is obtained from variation in concentration respective to its initial value $\Delta c_{i}\left( z,t \right)= c_{i}\left( z,t \right)-c_{i}\left( z,t=0 \right)$.

The RI increments, d*n/*d*c* are estimated from reported values^1,^^[2]^ assuming equal contribution of each ion, meaning (d*n/*d*c*)_K_ = 0.5 (d*n/*d*c*)_SO4_ = 0.01 RIU M^-1^.

For FcMeOH and FcMeOH^+^ the RI increments dn/dc were not directly available and were estimated from typical d*n*/d*c* values of aromatic molecules^[3]^, and a value of 0.05 RIU M^-1^ was used for FcMeOH. Noteworthy, the model does not require the precise knowledge of these absolute values but rather of the difference between d*n*/d*c* for the reduced and oxidized counterparts, and the optical response can then be reproduced whatever the absolute value of d*n*/d*c* for FcMeOH.

The cut-off and SPR wavelength shifts are evaluated as:

$\Delta\lambda_{cut-off}=m\int_{0}^{\infty} \Delta n(z) exp\left( -\frac{z}{l_{d}} \right)\frac{dz}{l_{d}}$	(SI7)

$\Delta\lambda_{\mathrm{SPR}}=m\int_{0}^{\infty} \Delta n(z)\exp\left( -\frac{z}{l_{d}} \right)\frac{dz}{l_{d}}+\kappa_{\sigma}\Delta Q+\sum_{i} \kappa_{\Gamma,i}\Delta\Gamma_{i}(t)$	(SI8)

Here *m* is the bulk sensitivity (577 nm RIU⁻¹), and *l*_d_ is the mode-specific evanescent-field penetration depth (shorter decay length for SPR, and longer for the cut-off mode, respectively taken as 400 nm and 1.5 µm). $\Delta Q$ is the change in double-layer charge density, and $\Delta\Gamma_{i}$the change in surface coverage of adsorbed species. The coefficients $\kappa_{\sigma}$ and $\kappa_{\Gamma,i}$ are effective interfacial optical sensitivities adjusted to reproduce the measured amplitudes of DLC and adsorption-induced shifts.

The model reproduces (i) the absence of cut-off response for pure DLC, (ii) the similar but weaker cut-off shifts for FcMeOH redox (Fig. S8b), and (iii) the larger, surface-enhanced SPR shifts in all cases, supporting the interpretation of SPR-TFBG as simultaneously sensitive to near-surface bulk gradients and interfacial adsorption/charge, while the cut-off mode mainly reports the former.

The overall model is available upon request.

**Fig. S8. a** Modelled cyclic voltammograms of the SPR-TFBG in 0.1 M K_2_SO_4_, showing increased adsorption at slower scan rates as detected and modeled through a decrease in SPR shift amplitude (experimental data shown in Fig. S3b). **b** Modelled SPR and cut-off responses for FcMeOH oxidation as in Fig. S9c. **c-g** Sensitivity of the model to characteristic parameters. **c** Effect of the sulphate adsorption/desorption characteristic time (40, 100, 400, 1000 s) and **d** relative coverage at pzc ($\theta_{\mathrm{pzc}}$= 0, 0.1, 0.5, 0.7, 1) on the relative SPR shift; see equations SI4 and SI5. In **c,** $\theta_{\mathrm{pzc}}$= 0.1, $\Gamma_{\max}$ = 0.3 nmol cm^-2^ and $c$= 0.5 M; in **d,** $\tau_{\mathrm{app}}$= 400 s, $\Gamma_{\max}$ = 0.3 nmol cm^-2^ and $c$ = 0.5 M. **e-h** Effect of FcMeOH **e** desorption or **f** adsorption rates and **g, h** diffusion coefficient on the relative SPR shift: **e** $k_{des,Fc}$ = 10, 1, 10^-1^, 10^-2^, 10^-3^, 10^-4^ s^-1^, $k_{ads,Fc}$ = 10^-1^ M^-1^ s^-1^; **f** $k_{ads,Fc}$ = 10^4^, 10^3^, 10^2^, 10, 1, 10^-1^, 10^-2^ M^-1^ s^-1^, $k_{des,Fc}$ = 5$\times$10^-3^ s^-1^; **g, h** *D*_Fc_ = 7$\times$10^-6^, 7$\times$10^-7^, 7$\times$10^-8^,7$\times$10^-9^, 7$\times$10^-10^ cm^2^ s^-1^, with adsorption in **f** $k_{ads,Fc}$ = 10^-1^ M^-1^ s^-1^, $k_{des,Fc}$ = 5$\times$10^-3^ s^-1^, or no adsorption in **g**. At high desorption rate or a slow adsorption rate, i.e. when adsorption is disfavored, the response tends toward the DLC behavior **e, f**. Decreasing the diffusion coefficient and therefore diffusion length does not change significantly the SPR response when it is dominated by the surface effect **g**, but can reveal the effect of the diffusion layer thickness when adsorption is eliminated **h**.

**Fig. S9. a** Cyclic voltammograms of the SPR-TFBG in 5 mM ferrocenemethanol and 0.5 M K_2_SO_4_ at scan rates ranging from 10 mV s^-1^ (dark blue) to 50 mV s^-1^ (light blue). **b** Comparison of SPR (purple) and cut-off mode (red) wavelength shifts during successive redox cycling in 5 mM ferrocenemethanol and 0.5 M K_2_SO_4_ at scan rate of 10 mV s^-1^. **c** Optical response during potentiostatic measurements at 0.35 V and 0.5 V vs Ag/AgCl, separated by five minute rest period at OCV.

**Fig. S10.** **a** SPR wavelength shift during the oxidation and reduction of the fourth cycle of 5 mM ferrocenemethanol in 0.5 M K_2_SO_4_ at a scan rate of 10 mV s^-1^. Sigmoidal fits of the **b** oxidation and **c** reduction half-cycles.

**Fig. S11.** SPR response during potentiostatic measurements at 0.35 V and 0.5 V vs Ag/AgCl, separated by a five minutes rest period at OCV (top) and corresponding first derivative of the SPR shift ($d\Delta\lambda/dt$) (bottom); same solution as in Fig. S9.

**Fig. S12. a** Schematic of the processes at the Au/H_2_SO_4_ interface during the oxidation-reduction cycle: from double layer rearrangement to Au-OH/Au-O formation and reduction (regions I-V). **b** SPR (purple) and cut-off mode (red) wavelength shifts, with **c** the corresponding first derivative ($d\Delta\lambda/dt$) tracking the electrochemical current during CV in 0.5 M H_2_SO_4_ at 5 mV s^-1^.

**Fig. S13. a** First and second cycles with a gold coated TFBG fiber in 50 mM lithium bis(oxalato)-borate (LiBOB) in 0.2 M LiClO_4_/DME at 2 mV s^-1^ using lithium metal as both counter and reference electrodes, from 2.5 V to 0.45 V. **b** Comparison of the relative SPR and cut-off shift during the first cycle. The difference in sensitivity ca. 50, is consistent with that observed for FcMeOH oxidation or PB electrochemistry (see Fig. S18-21). **c** SPR wavelength shift (purple) and its **d** first derivative plotted together with the electrochemical current (black), showing distinct kinetic signatures for the three cathodic regimes (region I-III) during SEI formation. **e** Second and **f** third cycles shown for comparison.

**Fig. S14.** **a** Cyclic voltammogram of 50 mM LiBOB in 0.2 M LiClO_4_/DME recorded using gold-coated TFBG fiber at 3 mV s^-1^, with lithium metal as both counter and reference electrodes. The inset shows the corresponding current as a function of time, with markers indicating the optical spectra displayed in b. **b** Representative transmission spectra at selected stages of the cycle. **c** SPR wavelength shift plotted against time, extracted from b. **d** Bragg wavelength shift of TFBG core mode.

**Fig. S15.** SEM characterization of Au-coated fibers (500 nm and 100 nm) after cycling through Li-Au alloying window.

**Fig. S16. a** Galvanostatic lithiation and delithiation of an Au-coated fiber in 50 mM LiBOB in 0.2 M LiClO_4_/DME at 1.5 C rate. Changes of TFBG envelope recorded during first, **b** second and **c** third cycles of gold-lithium alloy formation.

**Fig. S17.** Rietveld refinement of XRD patterns of a 500 nm thick Prussian blue film on a gold coated glass slide and its corresponding structural model.

Based on the electrochemical charge passed during the reversible PB/PW conversion associated to ion insertion, the film thickness, $t_{\mathrm{film}}$ was calculated assuming 100% Faradaic efficiency using Equation SI9^34^:

$t_{\mathrm{film}}= \frac{Q_{\mathrm{ox}}N_{A}d^{3}}{4FS}$	(SI9)

where $Q_{\mathrm{ox}}$ is the measured charge during oxidation, $N_{A}$ is Avogadro constant (6.022·10^23^ mol^-1^), $d$ is the lattice constant of the PB unit cell and the factor 4 accounts for the number of electrochemically active iron atoms per unit cell, $F$ Faraday’s constant (96485 C mol^-1^) and $S$ the geometric surface area (0.1445 cm^2^). Using a stored charge of 105 μC, the resulting film thickness was calculated to be 11.6 nm.

**Fig. S18. a** SPR shift (purple) and exchanged charge (black) during ion insertion and extraction in the first electrochemical cycle of electrodeposited Prussian blue at 10 mV s^-1^ in 0.25 M K_2_SO_4_ (pH 3.5). **b** Correlation between charge and SPR shift over the full cycle. **c** Boltzmann fit of the SPR shift and charge during ion insertion. **d** Boltzmann fit of the SPR shift and charge during ion extraction.

**Fig. S19. a** SPR (purple) and cut-off (red) mode shift variation (in pm s^-1^) during electrochemical cycling with ion insertion and extraction in electrodeposited Prussian blue at 5 mV s^-1^ in 0.25 M K_2_SO_4_ (pH 3.5). **b** Corresponding wavelength shift variation at 10 mV s^-1^ in 0.7 M NaPF_6_ in propylene carbonate (PC).

**Fig. S20. a** SPR wavelength shift (purple) and exchanged charge (black) during the first electrochemical cycle of Na⁺ insertion and extraction in electrodeposited Prussian blue, recorded at 10 mV s^-1^ in 0.7 M NaPF_6_ in propylene carbonate (PC), using sodium metal as both reference and counter electrodes. **b** Correlation between SPR shift and exchanged charge over the full cycle. **c** Boltzmann fits of SPR shift and charge during Na⁺ insertion. **d** Boltzmann fits of SPR shift and charge during Na⁺ extraction.

**Table 1. Comparison of operating principles and key sensing metrics for fiber Bragg gratings (FBGs), tilted fiber Bragg gratings (TFBGs) and gold-coated SPR-TFBGs.** Values in the SPR-TFBG column are from this work unless noted; FBG and TFBG entries are representative figures from the literature. DLC, double-layer charging; SEI/CEI, solid/cathode electrolyte interphase; RIU, refractive index unit.

| **Metric** | **FBG ^4^** | **TFBG ^5^** | **SPR-TFBG (this work)** |
| --- | --- | --- | --- |
| **Operating principle** | Reflection-mode core Bragg resonance | Transmission-mode cladding-mode resonances | Cladding modes coupled to surface plasmon resonance (≈50 nm Au film) |
| **Primary measurands** | Temperature, strain, pressure | Surrounding refractive index, concentration, composition, turbidity | Interfacial charge, adsorption, redox, film growth; plus bulk refractive index |
| **Probing depth** | Core-confined (no external field) | Cladding evanescent field, ≈ 0.8–1.5 µm | SPR ≈ 0.4 µm (interface) and cut-off ≈ 0.8–1.5 µm (bulk) |
| **Interfacial selectivity** | None | Limited (bulk-dominated) | High - plasmonic field isolates first nanometers; 10-100x improved interface/bulk contrast |
| **Quantitative sensitivity** | Temperature: ~ 10 pm °C^-1^ ; Strain : ~ 12 pm µε^-1^ | Bulk RI (mode-dependent - amplitude, envelope area, single-mode, or cut-off tracking) | 577 nm RIU^-1^ (R^2^ > 0.999); picometer λ-resolution 🡪 ≈ 10^2^ pg cm^-2^; 0.7-30 pm µC^-1^ (charge resolved) |
| **Working-electrode function** | No – passive optical element | No – passive optical element | Yes - Au coating acts as electrode and optical probe simultaneously |
| **Electrochemical information** | None (physical sensing only) | Bulk electrolyte chemistry | DLC, ad/desorption, SEI/CEI, redox, plating/alloying, intercalation, electrodeposition |
| **Key advantage** | Robust, multiplexable, EMI-immune, low-cost | Surrounding-medium sensitivity; built-in Bragg reference for ratiometric readout | Multiscale optical–electrochemical operando interfacial diagnostics; simultaneous electrode/optical dual-function |
| **Main limitation** | No chemical or ionic information | Limited interfacial selectivity; bulk-dominated response; weak surface sensitivity | P-polarization control; Au thickness/conformality reproducibility; irreversible films distort single-resonance tracking |

**Practical guidance towards the use of SPR-TFBG for electrochemical sensing**

1. *An optical metric for electrochemistry*

We quantify SPR-TFBG responses in picometers (pm), the natural unit for wavelength shifts in spectroscopy. With our bulk refractometric calibration, Δ*λ*_SPR_ converts to effective refractive index change Δ*n*_eff_ via Δ*λ*_SPR_ = *S_λ_* Δ*n*_eff_, with *S_λ_* ≈ 577 nm RIU^−1^. Thus, a single-pm shift corresponds to Δ*n*_eff_≈1.7×10^−6^ RIU. This level of sensitivity would quickly register even modest local heating (e.g., Δ*n*_eff_ = -10^-4^ per 1 K in many liquids, corresponding to a 57 pm blue-shift). Recognizing then that small thermal/strain drifts can also shift resonances, it is crucial for those experiments to minimize bending/stretching and stabilize ambient thermal conditions. When electrochemically induced, resonance shifts scale with charge: Δ*λ*_SPR_ ∝ Δ*Q*, enabling quantitative interpretation in pm per µC. The magnitude depends on the process; a pm-per-charge readout reflects optical mass/thickness of material accumulated or depleted at the interface per charge injected. It consistently highlights SPR’s superior sensitivity over cladding cut-off modes for probing DLC, film formation, or solid-phase conversion. The linear Δ*λ*_SPR_ - charge relationship provides "optical voltammograms": the time derivative ($d\Delta\lambda/dt$) overlaid with the electrochemical current provides mechanistic insights into interfacial electrochemical transformations.

2. *Surface versus bulk sensitivity: when does the SPR mode dominate?*

The SPR signal is dominated by interfacial charge and adsorption/desorption whenever the active chemistry is confined within the first few nanometers of the electrode (double-layer charging, SEI/CEI growth, electrodeposition, immobilized-film redox, plating), since these processes fall inside the plasmonic field, but contribute negligibly to the larger cut-off probing volume. Refractive-index changes in the solution contribute significantly when they extend across an appreciable fraction of the penetration depth, *l*_d_ (uniform electrolyte exchange, or a diffusion layer thickness *δ* comparable to *l*_d_). This could occur during uniform electrolyte exchange, concentration polarization, or solution-phase redox conversion. In this case, SPR and cut-off modes should respond with comparable amplitude, as observed in the bulk K_2_SO_4_ calibration. As a first approximation, the bulk calibration gives a sensitivity for K_2_SO_4_ (which should be evaluated for all probed species) of ca. 577 nm RIU⁻¹ for both modes. A uniform electrolyte concentration change of 1 mM, assuming a typical refractive-index increment of 0.015 RIU M⁻¹, would give a corresponding change in RI of ca. 1.5 $\times$10^-4^ RIU and therefore produce a wavelength shift of ca. 9 pm in both SPR and cut-off modes. A larger 0.1 M concentration change would correspond to ca. 0.9 nm variation, again in both channels.

When the diffusion layer extends far beyond the penetration depth (*δ* ≫ *l*_d_), as for FcMeOH, the concentrations within the first micrometer can be treated as quasi-uniform. The FcMeOH/FcMeOH^+^ conversion therefore should give a measurable common bulk RI change contribution in both modes. Any SPR response in excess of the cut-off (bulk) contribution then reflects an additional interfacial process such as adsorption/desorption at the Au surface.

3. *Back-of-the-envelope quantification*

The observed ~ 4.75 pm µC^-1^ (≈ 0.5 pm per µC cm^-2^) for DLC implies sensitivity to ~ 10 pmol cm^-2^ of ions, i.e. roughly one ion per 4 nm × 4 nm of surface. In organic electrolyte, interfacial SEI formation, Li underpotential deposition and Au alloying are resolved with < 10 pm sensitivity, corresponding to < 50 pmol cm^-2^ or ~ 0.04 Li per Au surface atom, exceeding the sensitivity of direct mass measurements such as EQCM.

4. *Temporal resolution*

The temporal resolution is set by the spectral acquisition interval (~ 5 s per spectrum in this study), which is a property of the specific optical interrogator used rather than an intrinsic limit of the method; faster tunable-laser and spectrometer-based systems reduce this to the sub-second or millisecond range and would extend the accessible temporal window accordingly. With the present instrumentation, a process is reliably resolved when its characteristic timescale is appreciably longer than ~ 5 s, such that the transition is sampled by multiple spectra; processes evolving at or below ~ 5 s are captured only in time-averaged or integrated form. Three regimes follow.

**(i) Reliably resolved (*τ* ≫ 5 s).** The cyclic voltammetry here was performed at 2-50 mV s^-1^, corresponding to tens to hundreds of seconds per sweep, so that each sweep is sampled by tens of spectra. In this regime the optical response faithfully tracks the electrochemistry, as verified by the close overlay of dΔ*λ*/d*t* with the measured current. This includes the slow interfacial adsorption/desorption dynamics quantified here (tens of seconds; e.g. ~ 3×10^-3^ s^-1^ at the pzc), minute-scale OCV relaxations, SEI growth over successive cycles, and the Prussian blue PB/PW(/BG) phase transitions, whose sigmoidal transition times (~4-10 s) are well sampled.

**(ii) Near the resolution limit (*τ* ≈ 5 s).** The fastest process we resolve is the FcMeOH redox transition, with a measured transition time of ~ 3 s, close to the ~ 2.5 s expected for a Nernstian system at this scan rate. Here the optical readout captures the overall envelope of the transition but not its fine temporal structure, placing it at the practical boundary of our current acquisition rate, and precisely the regime in which a faster interrogator would provide immediate benefit.

**(iii) Averaged or missed (*τ* < 5 s).** With the present instrument, faster phenomena, RC-limited double-layer charging (sub-second), fast electron-transfer kinetics, and any transient occurring within a single acquisition window, are not resolved as time-dependent events and contribute only through their integrated or steady-state effect; accessing them would require the higher-speed interrogation noted above.

We further note that, for solution-phase processes, the measured response time can be governed not by the sensor or interrogator but by coupled mass transport, the migration and diffusion of counter-ions and electroneutral species that must accompany the process of interest. In such cases the apparent temporal limit reflects the electrochemical system rather than the optical acquisition, and depends on the medium (aqueous versus aprotic, viscosity, ionic association). A focused study combining faster interrogation with these mass-transport considerations across different media is a natural direction for future work; the present results establish the resolution achievable with standard ~ 5 s acquisition and the quantitative basis for extending it.

5. *Practical considerations and limitations*

In aqueous media, responses ranged from 1 pm µC^-1^ upward, often higher in K_2_SO_4_ than H_2_SO_4_, suggesting hydrated solute polarizability plays a large role. In non-aqueous electrolytes, (e.g., LiClO_4_ + LiBOB in DME), initial faradaic reactions showed small responses, whereas highly polarizable Li deposition/stripping gave substantially larger signals. More common Li-ion battery electrolytes consisting of EC-EMC blends exhibit surprisingly large non-linear variations in dielectric constant with composition and this would deserve careful considerations for maximizing the signal strength when optimizing SPR coupling and quantification. Nevertheless, the method is attractive for speciation and dissolution monitoring: increases in dissolved transition metals (Ni or Mn) can dramatically alter electrolyte polarizability, enabling concentration monitoring in separators or reservoirs without applied potentials. Lastly, for active intercalation electrodes, PB-PW-BG systems showed shifts up to 30 pm µC^-1^, suggesting ‘pm/µC’ quantitatively tracks ion insertion/extraction and offers a tool for optimizing coatings and microstructures critical for next-generation cathode materials.

**References**

1. 1. (a) Kanoufi, F. Electrochemistry and Optical Microscopy. In *Encyclopedia of Electrochemistry* (ed. Bard, A. J.) 1–80 (Wiley, 2021); (b) Li, M. *et al.* Accurate Determination of Ion Polarizabilities in Aqueous Solutions. *The Journal of Physical Chemistry B* **121**, 6416–6424 (2017); (c) Utterback, J. M. *et al.* Operando Label-Free Optical Imaging of Solution-Phase Ion Transport and Electrochemistry. *ACS Energy Letters* **8***,* 1785–1792 (2023)
2. .	Rard, J. A. *et al.* Mutual Diffusion Coefficients and Refractive Index Increments of K_2_SO_4_(aq) at 298.15 K from Rayleigh Interferometry. *Journal of Solution Chemistry* **50**, 1315–1334 (2021).
3. 3. Davis, T. M. & Wilson W. D. Determination of the refractive index increments of small molecules for correction of surface plasmon resonance data. *Analytical Biochemistry* **284**, 348–353 (2000)

   4. Chen, D. *et al.* Recent Progress in Lithium-Ion Battery Safety Monitoring Based on Fiber Bragg Grating Sensors. *Sensors* **23**, 5609 (2023)

   5. Han, X. *et al.* Operando monitoring of dendrite formation in lithium metal batteries via ultrasensitive tilted fiber Bragg grating sensors. *Light: Science & Applications* **13**, 24 (2024)
